# Bioinformatics in *Español*: expanding open, diverse, and collaborative science through SEH2Bioinfo

**DOI:** 10.1093/bioadv/vbag182

**Published:** 2026-08-06

**Authors:** Carla L Padilla Franzotti, Gabriel J Olguín-Orellana, Nicolas Palopoli, Sebastián Urquiza-Zurich, Adrian Garcia-Moreno, Sara Monzón, Mónica Cabrera-Pasadas, Irene Soler Sáez, Carla Perpiñá-Clérigues, Rafael J Puche Quiñonez, Jennifer Vélez Segura, Jose Abel Lovaco-Flores, Cleidy M Osorio Mogollón, Eva M Esquinas-Román, María C Miserendino, Pradeep Eranti, Ana Castillo-Orozco, Yesid Cuesta-Astroz, R Gonzalo Parra

**Affiliations:** Departamento de Ciencia y Tecnología, Universidad Nacional de Quilmes, Buenos Aires, B1876, Argentina; Consejo Nacional de Investigaciones Científicas y Técnicas (CONICET), Buenos Aires, C1425FQB, Argentina; Departamento de Farmacología, Facultad de Ciencias Biológicas, Universidad de Concepción, Concepción, 4030000, Chile; Departamento de Ciencia y Tecnología, Universidad Nacional de Quilmes, Buenos Aires, B1876, Argentina; Consejo Nacional de Investigaciones Científicas y Técnicas (CONICET), Buenos Aires, C1425FQB, Argentina; Advanced Center for Chronic Diseases (ACCDiS), Facultad de Ciencias Químicas y Farmacéuticas, Universidad de Chile, 8380493, Chile; Department of Oncology-Pathology, Science of Life Laboratory, Karolinska Institute, Solna, 171 77, Sweden; Bioinformatics Unit, Institute of Health Carlos III, Majadahonda, Madrid, 28222, Spain; Computational Biology Group, Life Sciences Department, Barcelona Supercomputing Center (BSC-CNS), Barcelona, 08034, Spain; Computational Biomedicine Laboratory, Principe Felipe Research Center (CIPF), Valencia, 46012, Spain; Computational Biomedicine Laboratory, Principe Felipe Research Center (CIPF), Valencia, 46012, Spain; Laboratorio de Fisiopatologia, Centro de Medicina Experimental, Instituto Venezolano de Investigaciones Cientifcas (IVIC), Altos de Pipe, 1204, Venezuela; Departamento de Ingeneria Industrial y Sistemas - Universidad Nacional de Colombia, Bogotá D.C., 111321, Colombia; Department of Genetic Engineering, Center for Research and Advanced Studies of the National Polytechnic Institute (CINVESTAV), Irapuato, 36821, Guanajuato, Mexico; Department of Cell and Molecular Biology, Ribeirao Preto Medical School, University of Sao Paulo, 14049-900, Ribeirao Preto, SP, Brazil; Liver Metabolism and Disorders Laboratory, Center for Research in Molecular Medicine and Chronic Diseases (CiMUS) - University of Santiago de Compostela (USC), 15706, Santiago de Compostela, A Coruña, Spain; Institute of Science and Technology Austria (ISTA), Klosterneuburg, 3400, Austria; Université Paris Cité, Inserm, HealthFex, Paris, F-75006, France; Research Institute of the McGill University Health Centre (RI-MUHC), Montreal, Canada; Escuela de Microbiología, Universidad de Antioquia, Ciudad Universitaria, Medellín, 050010, Colombia; Evolutionary Systems Biophysics Group, Barcelona Supercomputing Center, Barcelona, 08034, Spain

## Abstract

Linguistic inequality remains a silent barrier in contemporary science, particularly in rapidly evolving and interdisciplinary fields such as bioinformatics. This article examines how the Spanish-speaking community has responded to this gap through the creation of the Simposio de Estudiantes Hispanohablantes de Bioinformática y Biología Computacional (SEH2Bioinfo), a collective initiative that transforms language into a tool for inclusion and collaboration. Drawing on a historical review of the use of Spanish in scientific communication and an analysis of the two editions of SEH2Bioinfo (2022 and 2025), complemented by surveys of more than 1500 participants, we explore the effects of language on confidence, participation, and academic visibility. The findings show that Spanish can serve as a space for training and mentorship without compromising rigor, while simultaneously strengthening international networks and promoting open science. The growth of SEH2Bioinfo, its collaborative approach, and its intercontinental reach establish the symposium as a model for linguistic equity in scientific practice. Its experience demonstrates that expanding linguistic diversity in bioinformatics is not only possible but essential to building a global scientific community that is fairer, more connected, and representative.

## 1 Introduction

English has become the de facto lingua franca of contemporary science, facilitating global exchange but creating significant barriers for non-English-speaking researchers. This dominance results in a stark imbalance: approximately 98% of global scientific publications are in English (Ramírez-Castañeda 2020), a reality that fosters structural inequalities in access, participation, and recognition, particularly in underserved regions.

Despite being the native language of 520 million people and the third most used language on the Internet (Instituto Cervantes 2025), Spanish is significantly underrepresented in international scientific databases. Bibliometric studies show that Spanish-language works receive fewer citations outside their national contexts (Moskaleva and Akoev 2019), highlighting a gap between its demographic weight and its representation in global academic output.

In this article, we argue that addressing these linguistic barriers requires more than just translation; it necessitates a robust conceptual understanding of how linguistic communities operate. To this end, we first establish a historical and theoretical framework regarding the role of Spanish in scientific communication, with a specific focus on its impact within Bioinformatics and Computational Biology.

Building on this foundation, we present the Symposium for Spanish-speaking Students in Bioinformatics and Computational Biology (SEH2Bioinfo) as a concrete and validated case study. Organized by various Regional Student Groups (RSGs) of the International Society for Computational Biology Student Council (ISCB-SC), this symposium serves as a scalable model for how regional communities can strategically organize to complement English dominance, fostering international collaboration and enhancing the visibility of Latin American scientific communities.

## 2 Spanish as a vehicle for inclusion in science

### 2.1 Challenges of English dominance in science

Between the 15th and 17th centuries, scientists typically used their native languages for everyday discussions about their work, while Latin was used for writing and communicating with researchers from other countries (Fransen 2017). By the early 20th century, the international scientific community had adopted a multilingual model dominated by English, French, and German, with these three languages used relatively equally (Hamel 2013). Eventually, during the 1970s, English was adopted as the lingua franca of science due to several factors, such as the desire to standardize scientific knowledge beyond national and cultural borders, the historical influence of English-speaking countries in science and technology, and the post-war expansion of universities and scientific journals that used English as their primary language (Ammon 2001).

Having a predominant language in science facilitates international collaboration, knowledge exchange, and the advancement of research in an increasingly interconnected world. It also supports the internationalization of education and the development of professional skills in the global scientific community (Tardy 2004). The use of a single language such as English allows for a standardized scientific technical terminology, which prevents confusion caused by different terms in various languages and fosters mutual understanding among researchers from different regions and disciplines (Montgomery 2004). A single, common language for scientific knowledge enables the efficient sharing of findings and advancements.

While this shift in the language of science promoted global collaboration and knowledge standardization, it also created significant barriers for scientists whose native language is not English, especially those from underserved regions. For these researchers, understanding and producing scientific content in English is a demanding challenge in terms of time and cognitive effort. This can affect the clarity and accuracy of their research and limits their ability to communicate results effectively (Ramírez-Castañeda 2020). For example, non-native researchers often invest far more time writing and editing manuscripts, have language-related rejection rates 2.5× to 2.6× higher than native speakers, and are asked for many more revisions. They are also less likely to attend or present at international conferences, and tend not to publish their best work in their own languages (Amano *et al.* 2023). Moreover, differences in language proficiency can lead to comprehension difficulties, limited vocabulary, pronunciation problems, and obstacles to self-expression, which can undermine confidence, especially in young researchers or students from underrepresented institutions (Gilakjani and Sabouri 2016, Kim *et al.* 2019).

The overwhelming dominance of English in science has contributed to a significantly greater number of publications and events in this language, which are also perceived as more prestigious. This linguistic hegemony creates biases that marginalize knowledge produced in other languages, limiting our ability to accurately understand how natural and social systems function in regions such as Latin America, Africa, or Asia, where valuable knowledge is generated but often neither translated nor disseminated globally (Amano *et al.* 2016). These barriers disproportionately affect researchers who face challenges in language learning, including individuals with special educational needs (Ehrman 1996, Gilakjani and Sabouri 2016, Boyle *et al.* 2020), thus widening inequalities in access to and production of scientific knowledge. Moreover, the structural preference for research originating from English-speaking institutions reinforces asymmetries in professional development and restricts the participation of non-English-speaking scientists in key arenas of global science, such as high-impact publications, collaboration networks, international funding, or engagement with strategic actors in the science and technology sector (Ammon 2012). As a result, these dynamics hinder both the individual advancement and collective development of local scientific communities.

### 2.2 Historical perspectives: Spanish in scientific communication

Spanish has a rich albeit fluctuating history in scientific communication. In the late 19th and early 20th centuries, there were concerted efforts in Spain to elevate national science and communicate it in Spanish. A pivotal moment was the establishment of the Junta de Ampliación de Estudios e Investigaciones Científicas (JAE) in 1907, under the leadership of Nobel laureate Santiago Ramón y Cajal, who, together with his contemporaries (e.g. Blas Cabrera, Miguel Catalán, Severo Ochoa), recognized that disseminating scientific knowledge to the public was vital for the progress of Spain (López-Pérez and Olvera-Lobo 2017). They organized public lectures and conferences, underscoring the importance of communicating science in their native language. A landmark event was the visit of Albert Einstein to Spain in 1923, during which he gave a series of lectures that captured national attention. Although his talks were delivered in German, his native language, simultaneous interpretation was provided, and written Spanish versions were made to facilitate the understanding and dissemination of his ideas. This enthusiasm sparked greater interest in the exact sciences and underscored the value of high-profile scientific communication that, while initially foreign, was embraced and spread in Spanish (López-Pérez and Olvera-Lobo 2017).

However, this blossoming was cut short by the Spanish Civil War (1936–39) and the subsequent Francoist dictatorship (1939–75), which led to the dismantling of scientific institutions such as the JAE, the exile of numerous researchers, and a profound intellectual isolation (Claret 2025). During this period, much of the human and institutional capital necessary to sustain a strong and accessible scientific culture was lost. It was not until the 1960s and 1970s that efforts to reconnect science with society were reactivated, through international colloquiums and Ibero-American seminars on science journalism, as well as the creation of the Asociación Española de Comunicación Científica (AEC2) in 1970 (López-Pérez and Olvera-Lobo 2017). These actions marked a process of re-professionalization of science communication in Spanish toward the end of the regime.

During the 20th century, Latin America experienced a scientific history marked by exchange with European and North American centers. Albert Einstein’s tour through South America in 1925 had a similar impact as his visit to Spain 2 years earlier (Tolmasquim 2011, Gangui and Ortiz 2016). Many researchers from the region trained or collaborated with institutions from the Global North, which strengthened local capacities but also imposed pressure to publish in English as a path to gain international recognition. However, alongside this, relevant Spanish-language initiatives emerged aimed at outreach and strengthening national scientific communities. Examples include the Revista de la Academia Colombiana de Ciencias Exactas, Físicas y Naturales (Colombia 1936), the journal Interciencia (Venezuela, 1976), the journal Ciencia Hoy (Argentina, 1988), and the journal ¿Cómo ves? (Mexico, 1998) from UNAM, as well as the creation of research councils and scientific academies that promoted local publications. While Latin America is recognized as a pioneer in open access publishing, these journals have suffered from a limited, regional reach (Moutinho 2024). Overall, this process created a persistent duality: while Spanish remained the language for teaching and communication with non-specialized audiences, English consolidated as the prestigious language for high-impact scientific publishing, in line with the rise of English as the globally dominant language in science.

During the transition to the 21st century, studies identified structural factors that deepened the displacement of Spanish as a scientific language, including low social demand for science, limited institutional support for outreach, disinterest from traditional media, and unequal scientific literacy (López-Pérez and Olvera-Lobo 2017). These factors reinforced the perception that publishing in Spanish was less valuable, particularly in the natural sciences, and reduced both output and visibility. In recent decades, however, a renewed ecosystem has emerged, driven by both traditional outlets and digital platforms. Notable examples include collaborative blogs such as Cienciaes, masScience, and Divulgame in Spain; general-audience publishers and projects like Fondo de Cultura Económica in Mexico and El Gato y La Caja in Argentina; the news agency Agencia SINC (Spain); the regional professional network RedPOP (Latin America and the Caribbean); and books like Vida.exe (González *et al.* 2022, Argentina), which aim to bring bioinformatics closer to Spanish-speaking audiences.

In parallel, open-access infrastructures have helped reposition Spanish as an academic language: platforms including SciELO, Latindex, RedALyC, and AmeliCA have expanded publishing opportunities for early-career and underrepresented researchers across Ibero-America, while projects like the Helsinki Initiative on Multilingualism in Scholarly Communication advance principles to strengthen linguistic diversity. Building on this foundation, a dynamic, public-oriented circuit has emerged, encompassing initiatives across multiple countries in Latin America, such as Clubes de Ciencia and Nerdearla in Latin America, and Congreso Futuro in Chile, that broaden access to and participation in science.

Complementing these developments, regional and international networks are knitting together interdisciplinary communities in bioinformatics, biotechnology, and data science, notably Capacity Building for Bioinformatics in Latin America network (CABANAnet), Allbiotech, Women in Bioinformatics and Data Science Latin America (WBDS LA), and R-Ladies. Within this ecosystem, the Latin American Conference on the Use of R in R&D (LatinR) has emerged as a fundamental trilingual space (Spanish, Portuguese, and English) that highlights the importance of the R programming language for regional research and software development. Additionally, the rOpenSci Champions Program has been instrumental in promoting open science by providing mentorship and increasing the participation of Latin American researchers in global research software communities. At the same time, initiatives such as MetaDocencia, RiaBio, and OpenCon prioritize inclusive scientific education and the creation of open educational resources in Spanish.

Taken together, these initiatives have established Spanish as a pivotal medium for education, scientific outreach, and regional collaboration. While English remains indispensable for global visibility, Spanish increasingly anchors open, locally grounded science, especially in bioinformatics, where open access to cutting-edge tools, networked collaboration to share opportunities and resources, and multilingual dissemination are critical to progress.

### 2.3 Structural and epistemic consequences of linguistic inequity

Beyond access and communication, the dominance of English in scientific publishing has profound structural and epistemic consequences. Scientific writing in English reflects specific traditions, argumentation styles, and disciplinary norms rooted in Anglo-American academia, which can marginalize knowledge systems, methodologies, and research agendas from other linguistic or cultural contexts (Moreno *et al.* 2012). This dynamic is part of a broader problem of linguistic marginalization and exclusion, also seen in social and legal spheres where the lack of recognition and legitimization of minority languages limits the full participation of their speakers (May 2003).

For Spanish-speaking researchers, this creates a tension between producing locally relevant knowledge and publishing in high-impact journals aimed at global audiences that are predominantly English-speaking and Eurocentric. This dynamic contributes to a form of epistemic colonization, where knowledge is only considered legitimate if produced or validated in English, which limits the visibility and credibility of research published in Spanish, even when addressing critical issues for the region, such as biodiversity, public health, or science education (Hultgren 2019). Moreover, infrastructure limitations, such as the underrepresentation of Spanish-language journals in international databases, reinforce this inequity, affecting funding, rankings, and academic careers. In this way, scientific participation becomes increasingly unequal, perpetuating the dominance of institutions with greater resources.

## 3 Spanish in bioinformatics and computational biology at a global stage

Bioinformatics and Computational Biology, broadly defined as the application and development of computational tools to analyze biological data, are by nature interdisciplinary and international disciplines that emerged in the late 20th century within predominantly English-speaking research environments. Yet, as these fields mature, there is a growing recognition of the need to teach and discuss them in other languages to broaden participation, build capacity globally and welcome scientific innovation and progress.

To advance this objective, several universities in Spanish-speaking countries have developed bioinformatics and computational biology programs taught primarily in Spanish. In Spain, in 2002, the first postgraduate program in bioinformatics was inaugurated: the Master’s in Bioinformatics and Data Science in Personalized Precision Medicine and Health, offered by the National School of Public Health at the Carlos III Health Institute. At the undergraduate level, the University of Talca (Chile) was a pioneer, launching its Bioinformatics Engineering program in 2003, the first in Latin America specifically designed for undergraduate training in this field. In 2006, the University of Entre Ríos (Argentina) established its Bachelor’s in Bioinformatics, further consolidating the regional expansion of academic offerings. Subsequently, the University of the Republic (Uruguay) launched its National Master’s in Bioinformatics in 2009, a multicenter effort to strengthen regional expertise. It was followed by the University of Costa Rica, which in 2011 created its Master’s in Bioinformatics and Systems Biology, and the National University of Colombia, which in 2012 introduced its Master’s in Bioinformatics. Most recently, in 2024, the National University of San Marcos (Peru) inaugurated its Master’s in Bioinformatics and Omics Sciences, thereby expanding the academic presence of the discipline across the continent.

In these and other similar programs, bioinformatics education is often bilingual; for instance, several Spanish universities reflected in the Guía de Formación en España en Bioinformática (Parra-Perez *et al.* 2021) offer bioinformatics degrees where course materials might be in English but complementary discussions and tutoring occur in Spanish, allowing students to grasp complex topics in their native language. In this way, such programs not only train students in cutting-edge biological computational analysis, but also cultivate Spanish vocabulary for bioinformatics concepts, helping to bridge the language gap. On the other hand, these initiatives also keep and emphasize the importance of English proficiency, promoting collaboration with leading research groups around the world, fostering the exchange of diverse perspectives and expertise and reaching a broader audience, thus increasing the visibility of their research.

Beyond formal degrees, there are ongoing efforts to translate key bioinformatics resources into Spanish. Volunteer-driven projects have tackled the translation of popular online courses and documentation for widely used bioinformatics tools. For example, segments of the Bioconductor project (an open-source software ecosystem for genomic data analysis in R) have Spanish translations or tutorials written by Latin American contributors. Similarly, training workshops within the Software Carpentry/Data Carpentry framework, which teach computational skills for data science, have been offered in Spanish. Additionally, digital libraries and Massive Open Online Course (MOOC) providers now release parallel editions in Spanish more routinely. Khan Academy and Coursera, for example, offer bioinformatics courses in Spanish, while newer platforms such as Genotipia, one of the sponsors of the 2nd edition of SEH2Bioinfo, delivers complete programmes in medical genetics entirely in Spanish, several of which are backed and certified by accredited higher-education institutions.

Nevertheless, several challenges persist. The rapid pace of updates in bioinformatics software and literature means community translations often lag, making the re-communication of findings into Spanish for teaching or outreach an additional step that is not always completed. A second obstacle is specialized terminology: when English labels are simply imported, students must learn concepts through opaque, non-descriptive names, increasing cognitive load. To mitigate this, some instructors use descriptive Spanish paraphrases (e.g. machine learning as “aprendizaje automatizado de patrones”), but achieving community-wide consensus on terminology takes time. Initiatives such as Enclave de Ciencia encourage adapting new concepts into Spanish rather than defaulting to English. However, translation alone is insufficient; effective understanding frequently requires contextualization, examples, datasets, and case studies drawn from local realities, that make the material genuinely meaningful to learners.

On the organizational front, Spanish-speaking bioinformaticians have formed networks to support one another and advance the field regionally. A significant example is the Iberoamerican Society of Bioinformatics (SoIBio), an international scientific society founded in 2009 with the aim of connecting research groups across Spain, Portugal, and Latin America. SoIBio provides a platform for collaboration and holds regular conferences (often branded as ISCB-Latin America meetings in partnership with the ISCB), where Spanish and Portuguese are commonly spoken alongside English (De Las Rivas *et al.* 2019). By fostering a sense of community, SoIBio encourages the sharing of training materials and research opportunities in Spanish. Additionally, CABANAnet has played a role in bioinformatics training with attention to language. Funded by the Global Challenges Research Fund and the Chan Zuckerberg Initiative DAF, CABANAnet has delivered workshops and fellowships across Latin America in three areas: communicable diseases, sustainable food production, and protection of biodiversity. While the scientific content has been universal, instructors often incorporated Spanish explanations and locally relevant case studies. The outcome has been a cohort of Latin American bioinformaticians who are comfortable operating in English but can also teach and mentor in Spanish, thus multiplying expertise within their home countries (Campos-Sánchez *et al.* 2024).

Socioeconomic disparities and language barriers reinforce one another in Latin American bioinformatics. Limited, uneven funding for core infrastructure, high-performance computing, secure storage, and specialist support pushes many institutions to rely on partnerships with better-resourced, often Anglophone, centers. These collaborations expand technical capacity, but because they typically operate in English, they can sideline Spanish in daily coordination and decision-making, concentrating communicative power in those with stronger English skills.

English proficiency in Latin America is strongly stratified by socioeconomic status, with high-quality instruction concentrated in private institutions and higher-income households, which in turn widens academic and professional gaps (Beltrão *et al.* 2021, Fernández *et al.* 2023). An example of this dynamic can be seen in Colombia, where among biology PhD students, higher socioeconomic status is associated with greater English proficiency and expanded professional opportunities (Ramírez-Castañeda 2020). Without robust Spanish-language pathways, training, documentation, workflows, and dissemination, the field risks excluding scientifically capable researchers whose main constraint is advanced English, thereby reproducing structural socio/economic inequities across underserved regions (Alhamami 2023, Turba *et al.* 2026).

### 3.1 The ISCB-SC landscape and current scenario

In 2004, the ISCB-SC was officially established, solidifying its position as the primary global community for students, postdocs, and early career researchers in the fields of bioinformatics and computational biology. To foster greater interaction among students and emerging scientists in the fields of bioinformatics and computational biology, the ISCB-SC created the Regional Student Groups (RSGs) program in 2006. This initiative was designed to facilitate the coordination of specific activities that cater to the needs of both students and young professionals in bioinformatics within their respective local communities. Due to its international reach, the RSGs program has catalyzed the establishment of multiple networks and collaborations among geographically distant communities (Shome *et al.* 2019).

**Figure 1:**
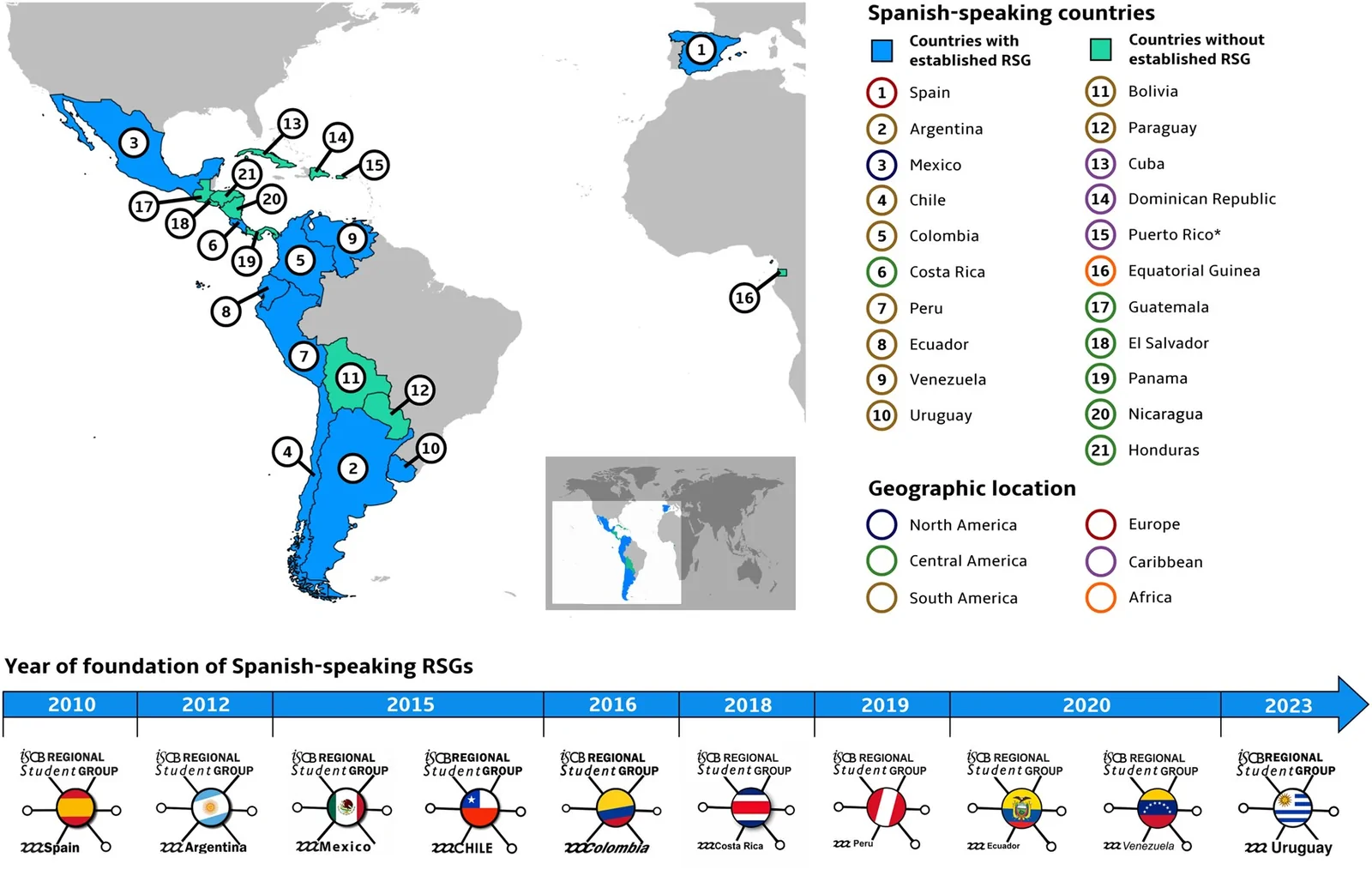
Evolution and geographic distribution of Spanish-speaking RSGs. The map (top) identifies Spanish-speaking countries with (blue) and without (green) established RSGs within the ISCB-SC. The timeline (bottom) illustrates the foundation years of the various groups, from 2010 to 2023. *Puerto Rico is included as part of the Spanish-speaking bioinformatics community due to the predominance of the Spanish language, although it is an unincorporated territory of the United States.

The ISCB-SC is expanding rapidly, with new RSGs forming worldwide; 10 are based in Spanish-speaking countries (Fig. 1), accounting for 40% of all regional groups (Eranti *et al.* 2023). Therefore, Spanish is the second most widely used working language on the web, after English. However, most flagship continental events organized by the ISCB-SC are conducted primarily in this language. While this promotes international collaboration and broader knowledge dissemination, it also creates a linguistic barrier for non-native speakers, particularly for students and early-career researchers.

The first Spanish-speaking RSG to be established was RSG-Spain in 2010, followed by RSG-Argentina in 2012 (Macintyre *et al.* 2013). In 2014, the Latin American leadership took the initiative to organize the first Latin American ISCB-SC Symposium (LA-SCS) (Parra *et al.* 2014), an event that has been held every 2 years since then (Parra *et al.* 2014, Monzon *et al.* 2017, Parisi *et al.* 2019, Castillo-Vilcahuaman *et al.* 2020). The LA-SCS marked a milestone in international communications in Latin America and served as a catalyst for the formation of new Spanish-speaking RSGs in the region. Since 2015, Spanish-speaking RSGs have expanded steadily (Fig. 1), and additional Spanish-speaking groups are expected in the coming years, further enlarging and strengthening this collaborative network.

Furthermore, since 2010, the Spanish-speaking community of the ISCB-SC has hosted a series of Student Symposia, for example, the inaugural meeting in Málaga (2010) as a pre-session of the Jornadas de Bioinformática co-organized by Portugal and the RSG-Northern Africa, followed by editions in Barcelona (2012) and Seville (2014) (Rivero *et al.* 2022). Subsequently, RSG-Argentina marked a regional milestone with the first Simposio Argentino de Jóvenes Investigadores en Bioinformática (SAJIB) in Buenos Aires (2016), the first national event organized by a Spanish-speaking RSG entirely in Spanish. It was carried out as a satellite of the annual conference of the Asociación Argentina de Bioinformática y Biología Computacional (A2B2C), conducted in English (Parra *et al.* 2016). Notably, in 2021, RSG-Ecuador and RSG-Venezuela jointly organized the ISCB-SC’s first cooperative, fully Spanish-language virtual event, expressly designed to meet the challenges posed by the COVID-19 pandemic (Ayala-Ruano *et al.* 2022).

All these activities highlighted the need and desire for collaborative events in languages other than English, aimed at facilitating the onboarding of students and early career researchers in the professional community and the practice of scientific meetings, and promoting the advancement of bioinformatics and computational biology in non-English-speaking nations, where ISCB and ISCB-SC have a presence.

### 3.2 Barriers and opportunities

The ISCB-SC is aware that language can open doors or quietly keep them shut. Surveys in Latin America reveal that a third of young biologists decline international conferences because English presentations are mandatory, and many spend sums equivalent to half a doctoral stipend just to have papers edited into flawless English (Ramírez-Castañeda 2020). Such figures underscore how the dominance of a single language magnifies pre-existing economic divides and sidelines promising scientists long before their ideas reach the peer-review stage. Yet the very structure of the Spanish-speaking RSGs demonstrates that inclusion need not wait for wider systemic change: it can begin in the native language of the students.

Inside the RSGs, early-career researchers have the opportunity to rehearse every facet of scientific life first in their native language. Because the audience shares cultural references and idioms, feedback is direct and confidence-building: presenters refine arguments without the cognitive load of translating on the fly. The environment thus becomes a laboratory for soft-skill development: volunteers learn to draft abstracts, chair symposia, field tough questions, negotiating sponsorships, writing press releases, and mentoring juniors, all while practising the rhetorical precision that science communication demands. Only after multiple iterations do many participants switch to English, armed with a polished story and stage presence that can withstand the scrutiny of an international conference room. In this way, Spanish is not a crutch but a scaffolding that lets ideas rise to full height before they are prepared for the global stage.

The benefits extend well beyond individual careers. Because RSGs talks are often streamed and later summarized, their scientific content has been able to re-enter classrooms and science outreach social networks across Latin America and Spain, showing school pupils, academics, and potential policy-makers that bioinformatics can also speak their language. Mentorship chains form naturally: senior members who have already leaped English-language conferences and journals guide newcomers through grant writing and conference abstracts, while still emphasizing the value of publishing locally relevant results in Spanish-speaking outlets. Thus, each RSG becomes both a springboard into the international ISCB network and a conduit back to the Spanish-speaking public sphere. From the ISCB-SC’s perspective, this recursive flow (local incubation, global presentation, local reinvestment) enhances scientific inclusivity where linguistic diversity enriches, rather than fragmenting the community leads to global integration.

## 4 SEH2Bioinfo: a model for inclusive and collaborative science in Spanish

### 4.1 Origins, objectives, and growth

SEH2Bioinfo is a unique international initiative that promotes the use of Spanish as a scientific language. Organized by ISCB-SC’s RSGs from Spanish-speaking countries, the event aims to overcome language barriers in science, specifically in computational biology and bioinformatics, and facilitate equitable access to knowledge in Spanish-speaking regions.

**Figure 2:**
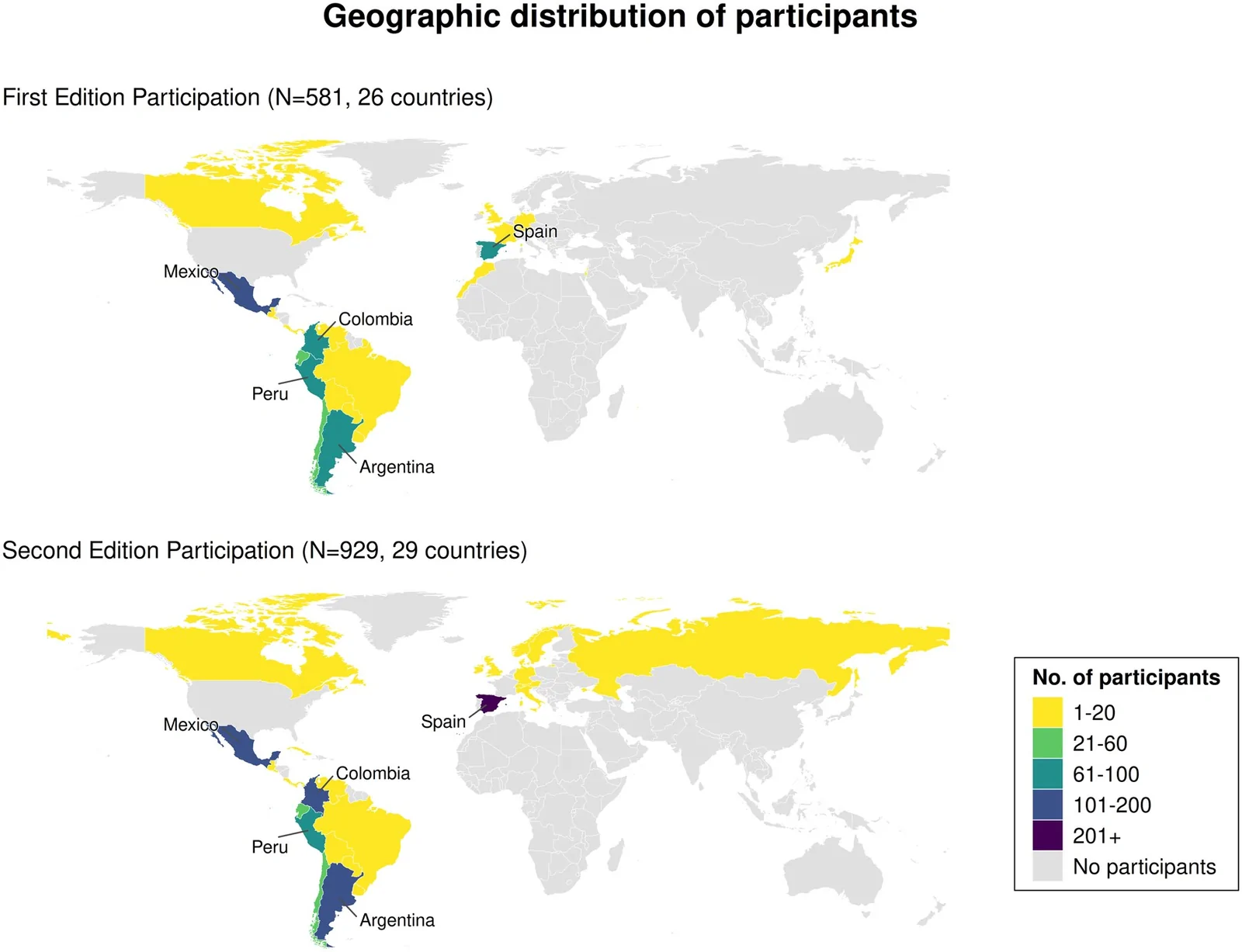
Geographic distribution of participants across SEH2Bioinfo editions. Data represent the first (top) and second (bottom) editions of the symposium. Country representation is based on institutional affiliation. The color scale categorizes the number of participants per country, with light gray indicating no participation. Countries with a significant volume of participation (n>60) in both editions are explicitly labeled.

The first edition, held in June 2022 (SEH2Bioinfo 2022), brought together 581 participants, becoming the largest multi-RSG event in the history of the ISCB-SC. Three years later, in April 2025, the second edition (SEH2Bioinfo 2025) gathered 929 attendees from Ibero-America, Europe, North America, and Asia, showing significant growth in both size and geographic reach (Fig. 2). The geographical distribution of participants in Fig. 2 also highlights the strategic importance of the Spanish-speaking scientific diaspora (Meyer 2001). While most attendees were based in Ibero-American institutions, the presence of researchers in non-Spanish-speaking countries, particularly in Europe, underscores the role of Spanish as a professional and emotional complement to English for scientists working abroad. However, the lack of participation from researchers in the USA was unexpected, given the significant number of Hispanic scientists in North American academic institutions. This absence suggests a gap in current outreach strategies and reinforces the need for targeted engagement with Hispanic scientific societies in non-Spanish-speaking countries. Strengthening these links will be crucial to positioning Spanish not as a substitute, but as a complementary tool that fosters collaboration and linguistic equity within the global scientific community. This progress reflects a growing global interest in scientific initiatives conducted in Spanish and highlights the symposium’s value as a model for linguistic inclusion and international collaboration. The diverse profile of participants, from undergraduate, master’s, PhD students to early-career researchers and professionals, demonstrates the potential of Spanish as an integrative tool for the new generation of scientists in computational biology and bioinformatics.

### 4.2 Format, activities, and organization

SEH2Bioinfo was held in a fully virtual and free format, facilitating direct interaction and networking between authors and attendees in an immersive online environment. The program included talks by keynote speakers (Table 1), as well as oral presentations, flash talks, and poster sessions delivered by students and early-career researchers, all in Spanish, thereby strengthening linguistic inclusion and scientific exchange.

**Table 1:** Summary of the main features of the 2022 and 2025 SEH2Bioinfo editions.

Symposium edition	First	Second
Organizing RSGs	Argentina, Spain, Chile, Mexico, Venezuela, Colombia, Peru, Ecuador, Costa Rica and Uruguay^a^	
Date	2 and 3 June 2022	1 and 2 April 2025
Registered participants	581	929
Oral presentations	14	22
Poster presentations	73	108
Represented institutions	163	183
Keynote speakers	Fátima Al-Shahrour (Spain) Francisco Melo (Chile) Patricia Melin (Mexico)	Alfonso Valencia (Spain) Cristina Marino-Buslje (Argentina) Alejandra Medina-Rivera (Mexico)
Sponsors	5	6

a RSG Uruguay joined as an organizer only for the 2025 edition.

During the first edition, the Mentorship Program in Bioinformatics in Spanish was launched, pioneering in its kind, with 86 mentees and 23 mentors from various Spanish-speaking and some non-Spanish-speaking countries, establishing the foundation for long-term interactions. In 2025, the “Your Presentation in 2 Minutes” contest was introduced to showcase research in a concise and accessible format. All submissions underwent peer review by a committee of PhD students and early-career researchers using a standardized rubric based on originality, methodological soundness, reproducibility, and relevance. Covered topics included machine learning, single-cell transcriptomics, functional genomics, evolutionary biology, structural biology, multi-omics analysis, and biological network modeling, with most participants being undergraduate or graduate students and early-career researchers aged 20–35.

Both editions incorporated playful and cultural activities, such as a themed escape room and a quiz about Spanish-speaking countries, fostering an egalitarian and collaborative environment while strengthening the shared cultural identity of the community. In the second edition, Spotify playlists were curated for each Spanish-speaking country, allowing participants to explore the musical diversity of the countries involved, find inspiration during work sessions, and reinforce cultural and emotional connections.

SEH2Bioinfo received support from scientific societies and private sponsors, including ISCB-SC, the Spanish Instituto Nacional de Bioinformática (INB ELIXIR-ES), the Sociedad Chilena de Bioinformática (SCB), A2B2C, and businesses: Dreamgenics, Genotipia, and Vakmo Technologies. This support highlights that collaboration between scientific entities and the private sector is key to sustaining inclusive, high-quality events, strengthening young scientists’ connections with international networks, and legitimizing the symposium’s impact within the Spanish-speaking scientific ecosystem.

SEH2Bioinfo was collaboratively organized by the active Spanish-speaking RSGs (Table 1), with specialized subcommittees working through digital platforms. Inclusive communication, intercultural awareness, and teamwork strategies were implemented to manage cultural diversity and participants’ varying levels of experience. Although the events were conducted entirely in Spanish, communication with the ISCB-SC Executive Team and external collaborators was carried out in English, adopting an adaptive bilingual approach. Official channels and social media accounts published content in both languages to broaden visibility, while technical aspects such as connectivity, platform compatibility, and audience management were addressed through rehearsals, technical support, and contingency planning.

Overall, Table 1 summarizes the evolution of the symposium between 2022 and 2025, showing how transatlantic collaboration among Spanish-speaking countries, with their linguistic variants and communication styles, together with differences in the academic levels of the organizing committee, event management experience, and increasing participant engagement, contributed to consolidating SEH2Bioinfo as a reference in bioinformatics in Spanish.

### 4.3 The challenge of fostering participation in bioinformatics

**Figure 3:**
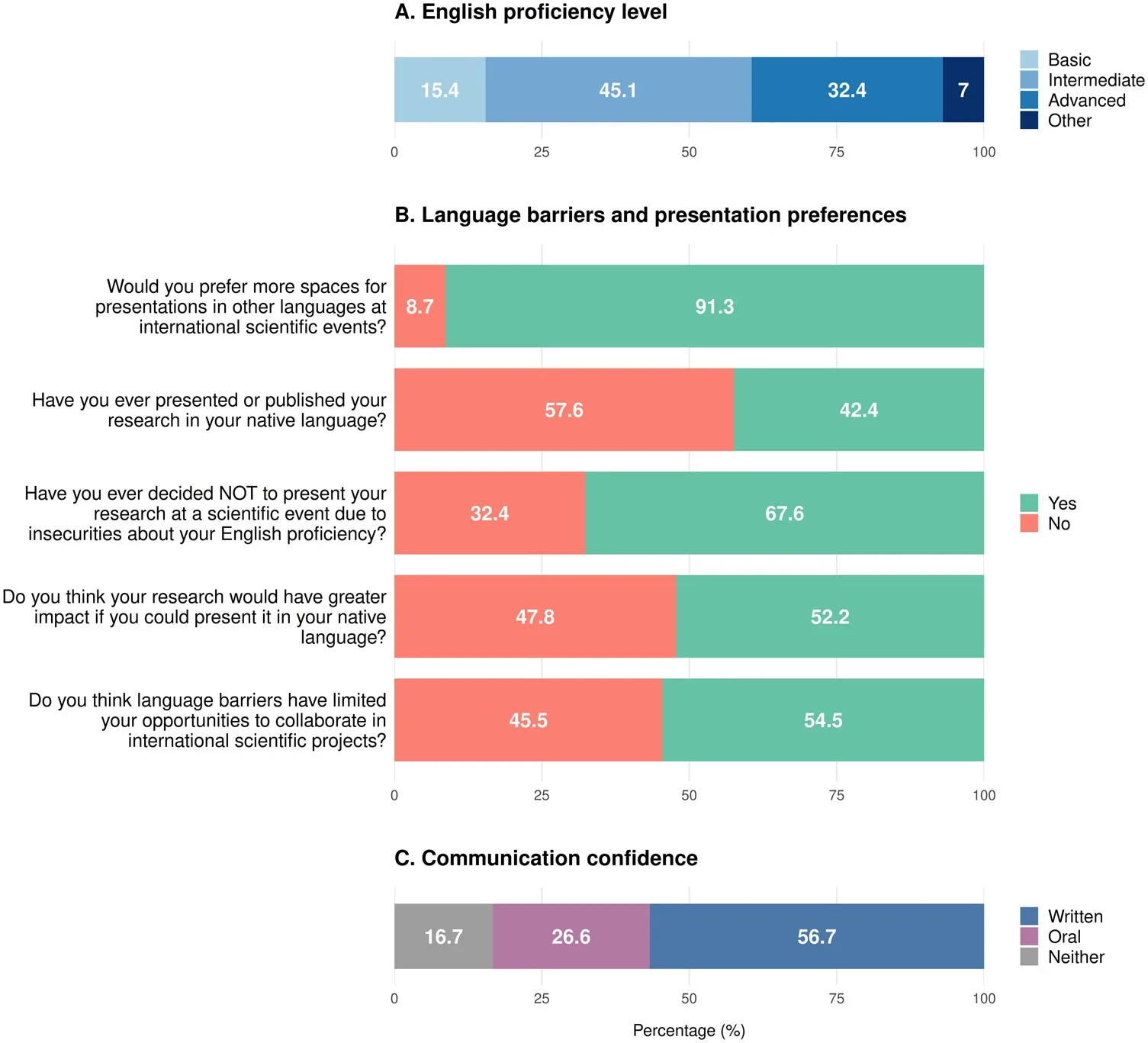
Percentage analysis of language barriers and self-reported English proficiency. Data were collected through surveys conducted during the first two editions of the SEH2Bioinfo event. (A) Distribution of English proficiency levels among participants. (B) Impact of language barriers on academic career paths and presentation preferences at international venues. (C) Comparative confidence levels based on communication mode. All values are expressed as percentages of the total sample (N=1510).

From surveys conducted during the two editions of SEH2Bioinfo, with a total of 1510 participants (Fig. 3), significant data emerge regarding the language barriers faced by the Spanish-speaking community of bioinformaticians and computational biologists. In the current scenario, the figure shows a community operating under English dominance but with uneven proficiency: roughly 61% of respondents place themselves at basic/intermediate levels, while only about 39% report advanced/near-native ability. This gap carries clear consequences. About 68% have avoided presenting at scientific events due to insecurities about their English, and 55% feel language barriers have limited international collaborations. A slim majority (52%) believe their work would have a greater impact if presented in their native language, yet only 42% have actually presented or published in it.

Preferences are unambiguous. An overwhelming 91% want more spaces for non-English presentations at international meetings. When they must use English, confidence skews to text: 57% feel more comfortable writing than speaking (27%), with 17% confident in neither, highlighting oral communication as a particular pain point.

Taken together, the data point to the necessity of practical, near-term interventions like multilingual tracks and poster sessions; bilingual abstracts and slides; live captioning/interpretation; options to present in Spanish with English materials; and coaching for in-public presentations, that would reduce self-exclusion, broaden collaboration, and better align dissemination with the community’s linguistic reality. SEH2Bioinfo serves as a concrete agent of change by implementing these measures. As an inclusive forum where participants can communicate clearly and confidently in Spanish, the symposium reframes language as a facilitator rather than a barrier and models policies that larger meetings could adopt. In this way, it serves as immediate equity infrastructure and as a catalyst for a deep evolution of the scientific ecosystem.

### 4.4 Impact and projections for the SEH2Bioinfo

SEH2Bioinfo, in its both editions, has become a reference point in the Spanish-speaking bioinformatics community. Its impact is reflected in its steady growth, strengthening of a regional network of young scientists, and the active use of Spanish for training, communication, and scientific collaboration. Its geographic reach includes not only Latin America and Spain but also extends to native Spanish speakers living in non-Spanish-speaking regions. This initiative is also of great interest to institutions around the globe that are involved in pushing inclusive communication models. The participation of individuals from approximately 200 public and private institutions from various countries supports the legitimacy and international projection of the event.

Compared to other international bioinformatics conferences which often focus on global communities and require high registration fees, SEH2Bioinfo stands out for its emphasis on the Spanish language and virtual accessibility. While traditional events limit the participation of students from economically disadvantaged regions, the SEH2Bioinfo maintains a free model that removes economic barriers and promotes equity. With its interactive format, combining engaging activities and multiregional roundtables, it has established itself as an innovative event within the Spanish-speaking scientific community. Its growth and institutional diversity position it as a key platform for promoting an accessible, inclusive, and global science from this community in the years ahead.

The next edition of SEH2Bioinfo is planned for 2028, ideally in person to maximize networking and mentorship. The aim is to convene the most outstanding bioinformatics leaders from Spanish-speaking countries, from established PIs to students, particularly those who have been active promoters of equity and inclusion in the field. Special emphasis will be placed on elevating young leaders with the capacity to train a new generation of bioinformaticians grounded in scientific excellence, social responsibility, and cultural enrichment. The program is envisioned to combine high-level keynotes with hands-on training, mentoring clinics, and science policy dialogues, consolidating SEH2Bioinfo’s role as a catalyst for a more inclusive and globally connected Spanish-speaking bioinformatics community. To achieve these goals, the symposium intends to adopt practical linguistic equity strategies inspired by successful international communities. Following models such as PyLadies Global, we will evaluate the implementation of AI-driven live captioning and simultaneous translation tools as part of our technological infrastructure. These actions aim to foster “receptive multilingualism” (ten Thije and Zeevaert 2007), a framework that leverages mutual intelligibility to ensure that core technical content remains accessible to all attendees despite potential language barriers. While our primary focus remains on strengthening the Spanish-speaking network to promote linguistic equity in science, we do not rule out the possibility of incorporating multilingual formats (e.g. Spanish-Portuguese-English) for future in-person or hybrid editions, following successful benchmarks like the Allbiotech initiative in fostering Ibero-American scientific collaboration.

## 5 Future outlook: Spanish as an effective and inclusive scientific language

Spanish now stands at the threshold of a more prominent role in scientific exchange, with momentum strengthening across multiple fronts. Editors are quietly reshaping the publishing landscape: a growing number of journals and conferences encourage or require authors to add a Spanish lay summary or abstract to the traditional English text. Emerging Themes in Epidemiology, for example, already translates abstracts into multiple languages. Brazil’s SciELO and Mexico’s RedALyC have spent two decades building respected open-access catalogues (Packer *et al.* 2014, Becerril-García and Aguado-López 2019). Moreover, funding agencies are exploring new strategies to further enhance their prestige, ranging from supporting professional translations to incorporating regional citation metrics, with the goal of ensuring that publications in Spanish achieve an impact comparable to those in English (Vessuri *et al.* 2014, UNESCO 2021).

Technological progress promises to accelerate that cultural shift. Machine-translation systems tuned specifically to scientific prose are approaching a level where a paper written in English can be converted almost instantaneously into Spanish with discipline-appropriate terminology. Scientists might be able to submit a paper in Spanish to a non-Spanish journal and rely on automatic translations for peer-reviewing and publication. Pilot projects have already pooled translated preprints into open repositories, effectively crowdsourcing a multilingual corpus of science. In a not-too-distant future, conference speakers could address an audience in Spanish while an AI subtitle stream renders the talk in English (whichever language the participant prefers). Such tools redistribute the communicative burden: English speakers will soon read Spanish research in seamless translation, rather than expecting every Spanish-speaking scientist to master English first.

Governments and institutions are lending formal weight to these trends. Spain’s Fundación Española para la Ciencia y la Tecnología (FECYT) has begun to require that grant deliverables include a plain-language summary in Spanish, and the agency funds outlets such as Agencia SINC to ensure that domestic research reaches the public in Spanish. Across Latin America, Semana de la Ciencia (Week of Science) festivals introduce schoolchildren to laboratory demonstrations in Spanish, nurturing a new generation confident in discussing STEM topics in their native language. On the multilateral stage, UNESCO’s 2021 Recommendation on Open Science explicitly calls for multilingual dissemination, and the International Science Council has echoed this stance, signaling that language equity is no longer a niche concern but part of mainstream science-policy doctrine (UNESCO 2021).

Community energy is amplifying these efforts. Early-career researchers treat bilingual outreach as a moral duty and a professional branding. Projects such as CABANAnet, SoIBio, and Scientia at Boise State show that volunteer translators and communicators can build high-impact platforms that normalize Spanish and Portuguese in health, agriculture, and bioinformatics. As these scientists move into editorial boards and funding panels, they bring the conviction that journal bilingualism and South-South collaborations should be standard practice. Networks such as Programa Iberoamericano de Ciencia y Tecnología para el Desarrollo (CYTED) already coordinate thematic clusters across Spain and Latin America (Saigí-Rubió *et al.* 2017); a future consortium might publish a landmark study on Amazonian biodiversity in side-by-side English and Spanish, setting a precedent for global scholarship written for local stakeholders.

Interdisciplinary and computational fields may reap the first dividends. Bioinformatics pipelines that process public-health data lose value if local epidemiologists cannot read the interface; developers have begun to add internationalization (a.k.a i18n) teams to translate software strings and documentation (Ouhbi *et al.* 2017). The culture of open-source fits neatly with linguistic inclusion: Volunteer programmers can translate and adapt tools as easily as they incorporate new features. Online education follows the same trajectory. Khan Academy, Coursera, Genotipia and Flyteek have markedly expanded their Spanish catalogues, sketching Bioinformatics MOOCs delivered from day one in Spanish (Ding *et al.* 2014). Meanwhile, the Spanish Wikipedia, with over one million entries, is enriched through edit-a-thons that recruit experts to enhance scientific pages (Sélem-Mojica *et al.* 2024).

These advances do not erase every obstacle. English will remain the currency of Nobel-level publication for the foreseeable future and producing dual-language content demands time, money, and sustained institutional support. The push for Spanish must avoid creating an insular silo; the ideal outcome is a bilingual scientist who can publish in English for global reach yet still engage peers and publics in Spanish. True equity must also recognize the importance of indigenous languages (e.g. Quechua, Aymara, Guaraní), which, in many Latin American countries, are as culturally and historically significant as Spanish (Hornberger 2006). Given the demographic weight of more than 500 million native speakers, rapid advances in AI-enabled translation (Mager *et al.* 2023), and the sustained energy of a new generation of researchers, Spanish is poised to move from the margins of scientific discourse to a complementary partnership with English. In this emerging multilingual landscape, Spanish contributes regional insight, cultural context, and broader public reach, making scientific knowledge more genuinely accessible to the communities it is meant to serve.

## 6 Conclusion

English will continue to play a central role in global scientific communication. Our analysis, however, suggests that this centrality need not preclude complementary, carefully designed spaces in other languages that broaden participation and improve training, particularly for early-career researchers in rapidly evolving, interdisciplinary fields such as bioinformatics. By situating the use of Spanish within a historical and contemporary landscape, and by examining two editions of the SEH2Bioinfo, we offer evidence that language-inclusive formats can coexist with, and ultimately strengthen, international engagement.

Across the 2022 and 2025 editions of the SEH2Bioinfo (581 and 929 attendees, respectively) and a survey of 1510 participants, we observed persistent language-related frictions that affect presentation rates, collaboration, and self-efficacy. The symposium’s design, Spanish as the working language, peer-reviewed abstract selection, structured mentoring, and interactive networking, appears to mitigate some of these barriers while preserving scholarly standards. In practical terms, the SEH2Bioinfo functions both as near-term equity infrastructure: (i) a venue where participants can think and present in their strongest language; and (ii) as a bridge to global fora, through bilingual materials and professional development activities.

The implications extend beyond a single event or language. In bioinformatics, where applications in public health and agri-food systems depend on local implementation, multilingual training, documentation, and the use of case studies can enhance the relevance and adoption of tools and methodologies. The SEH2Bioinfo model, which combines the use of the local language for learning and community building with dissemination in English, could be applied to other contexts and complement open science initiatives, capacity-building efforts, and South-South collaboration.

With appropriate caution, we suggest several modest, evidence-informed steps for Spanish-speaking institutions and societies to consider: multilingual abstracts, tracks and posters sessions; live captioning or interpretation; options to present in Spanish with English-language materials; micro-grants for editing and translation; and recognition of bilingual mentorship and outreach. Such measures are low-cost relative to their potential to reduce self-exclusion and to align dissemination practices with the linguistic realities of researchers.

Taken together, our experiences support a complementary view of linguistic practice in science: English remains indispensable for global reach, while Spanish-language environments can enhance equity, training quality, and regional impact. We hope this evidence encourages journals, societies, and funders to pilot proportionate, data-driven multilingual measures that preserve the efficiencies of a common language while widening the circle of those able to participate in, and benefit from, contemporary bioinformatics.

Finally, we extend our sincere appreciation to the ISCB and the ISCB-SC for their continued commitment to fostering diverse, globally connected scientific communities. Their vision and support have made initiatives such as SEH2Bioinfo possible, demonstrating that inclusion and excellence can advance hand in hand.

## Supplementary Material

vbag182_Supplementary_Data

## Data Availability

The data underlying this article, including the abstract books for the first and second editions of the SEH2Bioinfo symposia, are available in Zenodo at https://doi.org/10.5281/zenodo.6603621 and https://doi.org/10.5281/zenodo.1,54,46,570, respectively.

## Supplementary material

Supplementary material available at *Bioinformatics Advances* online.
