# Correction: Bioinformatics in Español: expanding open, diverse, and collaborative science through SEH2Bioinfo

**DOI:** 10.1093/bioadv/vbag271

**Published:** 2026-09-28

This is a correction to: Carla L Padilla Franzotti, Gabriel J Olguín-Orellana, Nicolas Palopoli, Sebastián Urquiza-Zurich, Adrian Garcia-Moreno, Sara Monzón, Mónica Cabrera-Pasadas, Irene Soler Sáez, Carla Perpiñá-Clérigues, Rafael J Puche Quiñonez, Jennifer Vélez Segura, Jose Abel Lovaco-Flores, Cleidy M Osorio Mogollón, Eva M Esquinas-Román, María C Miserendino, Pradeep Eranti, Ana Castillo-Orozco, Yesid Cuesta-Astroz, R Gonzalo Parra, Bioinformatics in Español: expanding open, diverse, and collaborative science through SEH2Bioinfo, *Bioinformatics Advances*, Volume 6, Issue 1, 2026, https://doi.org/10.1093/bioadv/vbag182

The following change has been made to the originally published article. The erroneously omitted supplementary file, containing a Spanish translation of the article, has now been supplied to the paper.

