# Supplementary material for: Bioinformatics in Español: expanding open, diverse, and collaborative science through SEH2Bioinfo
Source: Bioinform Adv. 2026 Aug 6;6(1):vbag182. doi: 10.1093/bioadv/vbag182 (PMC13455081; doi:10.1093/bioadv/vbag182)
Supplement: Supplementary file 1 [file vbag182_supplementary_data.pdf]

Bioinformática en español: ampliando la ciencia abierta, diversa y colaborativa a través del SEH2Bioinfo

Carla L. Padilla Franzotti [1,2,†], Gabriel J. Olguín-Orellana [3,†,*], Nicolas Palopoli[1,2], Sebastián Urquiza-Zurich[4], Adrian Garcia-Moreno[5], Sara Monzón[6], Mónica Cabrera-Pasadas[7], Irene Soler Sáez[8], Carla Perpiñá-Clérigues[8], Rafael J. Puche Quiñonez[9], Jennifer Vélez Segura[10], Jose Abel Lovaco-Flores[11], Cleidy M. Osorio Mogollón[12], Eva M. Esquinas-Román[13], María C. Miserendino[14], Pradeep Eranti[15], Ana Castillo-Orozco[16], Yesid Cuesta-Astroz[17] and R. Gonzalo Parra [18,*]

[1] Departamento de Ciencia y Tecnología, Universidad Nacional de Quilmes, Buenos Aires, Argentina
[2] Consejo Nacional de Investigaciones Científicas y Técnicas (CONICET), Argentina
[3] Departamento de Farmacología, Facultad de Ciencias Biológicas, Universidad de Concepción, 4030000, Concepción, Chile
[4] Advanced Center for Chronic Diseases (ACCDiS), Facultad de Ciencias Químicas y Farmacéuticas, Universidad de Chile, Chile
[5] Department of Oncology-Pathology, Science of Life Laboratory, Karolinska Institute, Solna, Suecia
[6] Unidad de Bioinformática, Instituto de Salud Carlos III (BU-ISCIII), España
[7] Computational Biology Group, Life Sciences Department, Barcelona Supercomputing Center (BSC-CNS), Barcelona, España
[8] Computational Biomedicine Laboratory, Principe Felipe Research Center (CIPF), 46012, Valencia, España
[9] Laboratorio de Fisiopatología, Centro de Medicina Experimental, Instituto Venezolano de Investigaciones Científicas (IVIC), Venezuela
[10] Universidad Nacional de Colombia, Colombia
[11] Department of Genetic Engineering, Center for Research and Advanced Studies of the National Polytechnic Institute (CINVESTAV), Irapuato, México
[12] Ribeirão Preto Medical School, University of São Paulo, Brasil
[13] Center for Research in Molecular Medicine and Chronic Diseases (CiMUS), University of Santiago de Compostela (USC), Santiago de Compostela, A Coruña, España
[14] Institute of Science and Technology Austria (ISTA), Klosterneuburg, Austria
[15] Inserm, T3S, Université Paris Cité, F-75006, París, Francia
[16] Research Institute of the McGill University Health Centre (RI-MUHC), Montreal, Canadá
[17] Escuela de Microbiología, Universidad de Antioquía, Medellín, Colombia
[18] Evolutionary Systems Biophysics Group, Life Sciences Department, Barcelona Supercomputing Center (BSC-CNS), Barcelona, España
*Autores para correspondencia: gonzalo.parra@bsc.es; gabrielolguinorellana@gmail.com
†Estos autores contribuyeron por igual.

Resumen

La desigualdad lingüística sigue siendo una barrera silenciosa en la ciencia contemporánea, especialmente en campos interdisciplinarios y en rápida evolución como la bioinformática. Este artículo examina cómo la comunidad hispanohablante ha respondido a esta brecha mediante la creación del Simposio de Estudiantes Hispanohablantes de Bioinformática y Biología Computacional (SEH2Bioinfo), una iniciativa colectiva que transforma el idioma en una herramienta de inclusión y colaboración. A partir de una revisión histórica del uso del español en la comunicación científica y del análisis de las dos ediciones del SEH2Bioinfo (2022 y 2025), complementados con encuestas a más de 1500 participantes, exploramos los efectos del idioma sobre la confianza, la participación y la visibilidad académica. Los resultados muestran que el español puede funcionar como espacio de formación y mentoría sin comprometer el rigor, al tiempo que fortalece redes internacionales y promueve la ciencia abierta. El crecimiento del SEH2Bioinfo, su enfoque colaborativo y su alcance intercontinental consolidan el simposio como un modelo de equidad lingüística en la práctica científica. Su experiencia demuestra que ampliar la diversidad lingüística en bioinformática no solo es posible, sino esencial para construir una comunidad científica global más justa, conectada y representativa.

Palabras clave: diversidad lingüística, comunidades hispanohablantes, comunicación científica, ciencia abierta, bioinformática.

Introducción

El inglés se ha convertido en la lengua franca de facto de la ciencia contemporánea, facilitando el intercambio global pero generando barreras significativas para los investigadores no anglófonos. Esta dominancia produce un marcado desequilibrio: aproximadamente el 98% de las publicaciones científicas mundiales están en inglés (Ramírez-Castañeda, 2020), una realidad que fomenta desigualdades estructurales en el acceso, la participación y el reconocimiento, particularmente en regiones con menos recursos.

A pesar de ser la lengua materna de 520 millones de personas y el tercer idioma más utilizado en Internet (Instituto Cervantes, 2025), el español está significativamente subrepresentado en las bases de datos científicas internacionales. Estudios bibliométricos muestran que los trabajos en español reciben menos citas fuera de sus contextos nacionales (Moskaleva and Akoev, 2019), lo que pone de manifiesto una brecha entre su peso demográfico y su representación en la producción académica global.

En este artículo sostenemos que abordar estas barreras lingüísticas requiere más que una simple traducción; exige una comprensión conceptual sólida de cómo operan las comunidades lingüísticas. Con este propósito, establecemos primero un marco histórico y teórico sobre el papel del español en la comunicación científica, con especial atención a su impacto en la Bioinformática y la Biología Computacional.

A partir de esta base, presentamos el Simposio para Estudiantes Hispanohablantes de Bioinformática y Biología Computacional (SEH2Bioinfo) como un caso de estudio concreto y validado. Organizado por diversos Grupos Regionales de Estudiantes (RSGs) del Consejo Estudiantil de la Sociedad Internacional de Biología Computacional (ISCB-SC), este simposio funciona como un modelo escalable de cómo las comunidades regionales pueden organizarse estratégicamente para complementar el dominio del inglés, fomentando la colaboración internacional y aumentando la visibilidad de las comunidades científicas latinoamericanas.

2. El español como vehículo de inclusión en la ciencia

2.1 Desafíos del dominio del inglés en la ciencia

Entre los siglos XV y XVII, los científicos solían utilizar sus lenguas maternas en las discusiones cotidianas sobre su trabajo, mientras que el latín se empleaba para escribir y comunicarse con investigadores de otros países (Fransen, 2017). A comienzos del siglo XX, la comunidad científica internacional adoptó un modelo multilingüe dominado por el inglés, el francés y el alemán, con un uso relativamente equilibrado de estos tres idiomas (Hamel, 2013). Finalmente, durante la década de 1970, el inglés se estableció como lengua franca de la ciencia debido a varios factores: el deseo de estandarizar el conocimiento científico más allá de las fronteras nacionales y culturales, la influencia histórica de los países angloparlantes en ciencia y tecnología, y la expansión posbélica de universidades y revistas científicas que adoptaron el inglés como idioma principal (Ammon, 2001).

Contar con una lengua predominante en la ciencia facilita la colaboración internacional, el intercambio de conocimientos y el avance de la investigación en un mundo cada vez más interconectado. También apoya la internacionalización de la educación y el desarrollo de competencias profesionales en la comunidad científica global (Tardy, 2004). El uso de un único idioma como el inglés permite una terminología técnica científica estandarizada, lo que evita la confusión generada por distintos términos en diferentes lenguas y fomenta la comprensión mutua entre investigadores de diversas regiones y disciplinas (Montgomery, 2004). Un idioma común para el conocimiento científico posibilita compartir hallazgos y avances de manera eficiente.

Si bien este cambio en el idioma de la ciencia promovió la colaboración global y la estandarización del conocimiento, también generó barreras significativas para los científicos cuya lengua materna no es el inglés, especialmente para quienes provienen de regiones en desarrollo. Para estos investigadores, comprender y producir contenido científico en inglés supone un esfuerzo considerable en términos de tiempo y carga cognitiva. Esto puede afectar la claridad y precisión de su investigación y limita su capacidad para comunicar resultados de manera efectiva (Ramírez-Castañeda, 2020). Por ejemplo, los investigadores no nativos invierten con frecuencia mucho más tiempo en redactar y editar manuscritos, presentan tasas de rechazo relacionadas con el idioma entre 2,5 y 2,6 veces superiores a las de los hablantes nativos, y reciben muchas más solicitudes de revisión. Además, tienen menos probabilidades de asistir o presentar en conferencias internacionales, y tienden a no publicar sus mejores trabajos en sus propias lenguas (Amano et al., 2023). Asimismo, las diferencias en el dominio del idioma pueden derivar en dificultades de comprensión, vocabulario limitado, problemas de pronunciación y obstáculos para la autoexpresión, lo que puede minar la confianza, especialmente en investigadores jóvenes o estudiantes de instituciones subrepresentadas (Gilakjani and Sabouri, 2016; Kim et al., 2019).

El dominio abrumador del inglés en la ciencia ha contribuido a que exista un número significativamente mayor de publicaciones y eventos en este idioma, percibidos además como más prestigiosos. Esta hegemonía lingüística genera sesgos que marginan el conocimiento producido en otras lenguas, limitando nuestra capacidad para comprender con precisión cómo funcionan los sistemas naturales y sociales en regiones como América Latina, África o Asia, donde se genera valioso conocimiento que, con frecuencia, ni se traduce ni se difunde a nivel global (Amano et al., 2016). Estas barreras afectan desproporcionadamente a investigadores que enfrentan dificultades en el aprendizaje de idiomas, incluyendo personas con necesidades educativas especiales (Ehrman, 1996; Gilakjani and Sabouri, 2016; Boyle et al., 2020), lo que agudiza las desigualdades en el acceso y la producción de conocimiento científico. Además, la preferencia estructural por la investigación proveniente de instituciones anglófonas refuerza asimetrías en el desarrollo profesional y restringe la participación de los científicos no anglófonos en ámbitos clave de la ciencia global, como publicaciones de alto impacto, redes de colaboración, financiamiento internacional o la vinculación con actores

estratégicos del sector de ciencia y tecnología (Ammon, 2012). En consecuencia, estas dinámicas obstaculizan tanto el avance individual como el desarrollo colectivo de las comunidades científicas locales.

2.2 Perspectivas históricas: el español en la comunicación científica

El español tiene una historia rica, aunque fluctuante, en la comunicación científica. A finales del siglo XIX y comienzos del XX, hubo esfuerzos concertados en España para elevar la ciencia nacional y comunicarla en español. Un momento decisivo fue la creación de la Junta de Ampliación de Estudios e Investigaciones Científicas (JAE) en 1907, bajo el liderazgo del Premio Nobel Santiago Ramón y Cajal, quien, junto a sus contemporáneos (p. ej., Blas Cabrera, Miguel Catalán, Severo Ochoa), reconoció que divulgar el conocimiento científico al público era vital para el progreso de España (López-Pérez and Olvera-Lobo, 2017). Organizaron conferencias y charlas públicas, subrayando la importancia de comunicar la ciencia en su lengua materna. Un acontecimiento destacado fue la visita de Albert Einstein a España en 1923, durante la cual ofreció una serie de conferencias que captaron la atención nacional. Aunque sus charlas se impartieron en alemán, su lengua materna, se proporcionó interpretación simultánea y se elaboraron versiones escritas en español para facilitar la comprensión y difusión de sus ideas. Este entusiasmo despertó un mayor interés por las ciencias exactas y puso de relieve el valor de la comunicación científica de alto perfil que, aunque inicialmente extranjera, fue acogida y difundida en español (López-Pérez and Olvera-Lobo, 2017).

Sin embargo, este florecimiento fue interrumpido por la Guerra Civil Española (1936-1939) y la posterior dictadura franquista (1939-1975), que condujo al desmantelamiento de instituciones científicas como la JAE, al exilio de numerosos investigadores y a un profundo aislamiento intelectual (Claret, 2025). Durante este período se perdió gran parte del capital humano e institucional necesario para sostener una cultura científica sólida y accesible. No fue hasta los años sesenta y setenta que se reactivaron los esfuerzos por reconectar la ciencia con la sociedad, a través de coloquios internacionales y seminarios iberoamericanos sobre periodismo científico, así como con la creación de la Asociación Española de Comunicación Científica (AEC2) en 1970 (López-Pérez and Olvera-Lobo, 2017). Estas acciones marcaron un proceso de reprofesionalización de la comunicación científica en español hacia el final del régimen.

Durante el siglo XX, América Latina vivió una historia científica marcada por el intercambio con centros europeos y norteamericanos. La gira de Albert Einstein por América del Sur en 1925 tuvo un impacto similar al de su visita a España dos años antes (Tolmasquim, 2011; Gangui and Ortiz, 2016). Muchos investigadores de la región se formaron o colaboraron con instituciones del Norte Global, lo que fortaleció las capacidades locales pero también impuso la presión de publicar en inglés como vía para obtener reconocimiento internacional. Sin embargo, paralelamente surgieron iniciativas relevantes en español orientadas a la divulgación y al fortalecimiento de las comunidades científicas nacionales. Entre ellas se cuentan la Revista de la Academia Colombiana de Ciencias Exactas, Físicas y Naturales (Colombia, 1936), la revista Interciencia (Venezuela, 1976), la revista Ciencia Hoy (Argentina, 1988) y la revista ¿Cómo ves? (México, 1998) de la UNAM, así como la creación de consejos de investigación y academias científicas que promovieron publicaciones locales. Si bien América Latina es reconocida como pionera en la publicación de acceso abierto, estas revistas han tenido un alcance limitado y regional (Moutinho, 2024). En general, este proceso creó una dualidad persistente: mientras el español se mantuvo como lengua de enseñanza y comunicación con audiencias no especializadas, el inglés se consolidó como el idioma de prestigio para la publicación científica de alto impacto, en consonancia con el ascenso del inglés como lengua globalmente dominante en la ciencia.

Durante la transición al siglo XXI, los estudios identificaron factores estructurales que profundizaron el desplazamiento del español como lengua científica, entre ellos la baja demanda social de ciencia, el escaso apoyo institucional a la divulgación, el desinterés de los medios tradicionales y la alfabetización científica desigual (López-Pérez and Olvera-Lobo, 2017). Estos factores reforzaron la percepción de que publicar en español tenía menos valor, especialmente en las ciencias naturales, lo que redujo tanto la producción como la visibilidad. No obstante, en las últimas décadas ha emergido un ecosistema renovado, impulsado tanto por medios tradicionales como por plataformas digitales. Ejemplos destacados incluyen blogs colaborativos como Cienciaes, masScience y Divulgame en España; editoriales y proyectos de divulgación como el Fondo de Cultura Económica en México y El Gato y La Caja en Argentina; la agencia de noticias Agencia SINC (España); la red profesional regional RedPOP (América Latina y el Caribe); y libros como Vida.exe (González et al., 2022, ; Argentina), que buscan acercar la bioinformática a las audiencias hispanohablantes.

En paralelo, las infraestructuras de acceso abierto han contribuido a reposicionar el español como lengua académica: plataformas como SciELO, Latindex, RedALyC y AmeliCA han ampliado las oportunidades de publicación para investigadores en etapas tempranas y subrepresentados en toda Iberoamérica, mientras que proyectos como la Iniciativa de Helsinki sobre Multilingüismo en la Comunicación Académica impulsan principios para fortalecer la diversidad lingüística. Sobre esta base, ha emergido un circuito dinámico orientado al público general, que engloba iniciativas en múltiples países de América Latina, como Clubes de Ciencia y Nerdearla en Latinoamérica y Congreso Futuro en Chile, ampliando el acceso y la participación en la ciencia.

Complementando estos desarrollos, redes regionales e internacionales están tejiendo comunidades interdisciplinarias en bioinformática, biotecnología y ciencia de datos, entre ellas la red Capacity Building for Bioinformatics in Latin America (CABANAnet), Allbiotech, Women in Bioinformatics and Data Science Latin America (WBDS LA) y R-Ladies. Dentro de este ecosistema, la Conferencia Latinoamericana sobre el Uso de R en I+D (LatinR) ha emergido como un espacio trilingüe fundamental (español, portugués e inglés) que destaca la importancia del lenguaje de programación R para la investigación y el desarrollo de software regional. Asimismo, el Programa de Campeones de rOpenSci ha sido fundamental para promover la ciencia abierta, proporcionando mentoría y

aumentando la participación de investigadores latinoamericanos en comunidades globales de software de investigación. Al mismo tiempo, iniciativas como MetaDocencia, RiaBio y OpenCon priorizan la educación científica inclusiva y la creación de recursos educativos abiertos en español.

En conjunto, estas iniciativas han consolidado el español como un medio fundamental para la educación, la divulgación científica y la colaboración regional. Si bien el inglés sigue siendo indispensable para la visibilidad global, el español ancla cada vez más una ciencia abierta y localmente arraigada, especialmente en bioinformática, donde el acceso abierto a herramientas de vanguardia, la colaboración en red para compartir oportunidades y recursos, y la difusión multilingüe son críticos para el progreso.

2.3 Consecuencias estructurales y epistémicas de la inequidad lingüística

Más allá del acceso y la comunicación, el dominio del inglés en la publicación científica tiene consecuencias estructurales y epistémicas profundas. La escritura científica en inglés refleja tradiciones, estilos argumentativos y normas disciplinarias específicas, enraizadas en la academia angloamericana, que pueden marginar sistemas de conocimiento, metodologías y agendas de investigación provenientes de otros contextos lingüísticos o culturales (Moreno et al., 2012). Esta dinámica forma parte de un problema más amplio de marginación y exclusión lingüística, también observable en el ámbito social y jurídico, donde la falta de reconocimiento y legitimación de las lenguas minoritarias limita la plena participación de sus hablantes (May, 2003).

Para los investigadores hispanohablantes, esto crea una tensión entre producir conocimiento localmente relevante y publicar en revistas de alto impacto dirigidas a audiencias globales predominantemente anglófonas y eurocéntricas. Esta dinámica contribuye a una forma de colonización epistémica, en la que el conocimiento solo se considera legítimo si es producido o validado en inglés, lo que limita la visibilidad y la credibilidad de la investigación publicada en español, incluso cuando aborda problemas críticos para la región, como la biodiversidad, la salud pública o la educación científica (Hultgren, 2019). Además, las limitaciones de infraestructura, como la subrepresentación de revistas en español en bases de datos internacionales, refuerzan esta inequidad, afectando la financiación, los rankings y las carreras académicas. De este modo, la participación científica se vuelve cada vez más desigual, perpetuando el dominio de las instituciones con mayores recursos.

3. El español en la bioinformática y la biología computacional en el escenario global

La bioinformática y la biología computacional, definidas en términos amplios como la aplicación y el desarrollo de herramientas computacionales para analizar datos biológicos, son por naturaleza disciplinas interdisciplinarias e internacionales que surgieron a finales del siglo XX en entornos de investigación predominantemente anglófonos. Sin embargo, a medida que estos campos maduran, crece el reconocimiento de la necesidad de enseñarlos y discutirlos en otras lenguas para ampliar la participación, fortalecer capacidades a nivel global y favorecer la innovación y el progreso científico.

Para avanzar en este objetivo, varias universidades de países hispanohablantes han desarrollado programas de bioinformática y biología computacional impartidos principalmente en español. En España, en 2002, se inauguró el primer programa de posgrado en bioinformática: el Máster en Bioinformática y Ciencia de Datos en Medicina y Salud de Precisión Personalizada, ofrecido por la Escuela Nacional de Sanidad del Instituto de Salud Carlos III. A nivel de grado, la Universidad de Talca (Chile) fue pionera al lanzar su programa de Ingeniería en Bioinformática en 2003, el primero en América Latina específicamente diseñado para la formación de pregrado en este campo. En 2006, la Universidad de Entre Ríos (Argentina) creó su Licenciatura en Bioinformática, consolidando la expansión regional de la oferta académica. Posteriormente, la Universidad de la República (Uruguay) lanzó su Maestría Nacional en Bioinformática en 2009, un esfuerzo multicéntrico para fortalecer la experiencia regional. Le siguió la Universidad de Costa Rica, que en 2011 creó su Maestría en Bioinformática y Biología de Sistemas, y la Universidad Nacional de Colombia, que en 2012 introdujo su Maestría en Bioinformática. Más recientemente, en 2024, la Universidad Nacional Mayor de San Marcos (Perú) inauguró su Maestría en Bioinformática y Ciencias Ómicas, ampliando así la presencia académica de la disciplina en todo el continente.

En estos y otros programas similares, la educación en bioinformática es frecuentemente bilingüe; por ejemplo, varias universidades españolas reflejadas en la Guía de Formación en España en Bioinformática (Parra-Perez et al., 2021) ofrecen titulaciones en bioinformática donde los materiales de los cursos pueden estar en inglés, pero las discusiones complementarias y la tutoría se realizan en español, permitiendo a los estudiantes comprender temas complejos en su lengua materna. De este modo, tales programas no solo forman a los estudiantes en análisis computacional biológico de vanguardia, sino que también cultivan el vocabulario en español para los conceptos de bioinformática, contribuyendo a cerrar la brecha lingüística. Por otro lado, estas iniciativas también mantienen y enfatizan la importancia del dominio del inglés, promoviendo la colaboración con grupos de investigación líderes en todo el mundo, fomentando el intercambio de perspectivas y conocimientos diversos y alcanzando a una audiencia más amplia, aumentando así la visibilidad de su investigación.

Más allá de los títulos formales, existen esfuerzos continuos para traducir recursos clave de bioinformática al español. Proyectos impulsados por voluntarios han abordado la traducción de cursos en línea populares y documentación de herramientas de bioinformática ampliamente utilizadas. Por ejemplo, segmentos del proyecto Bioconductor (un ecosistema de software de código abierto para el análisis de datos genómicos en R) cuentan con traducciones al español o tutoriales escritos por colaboradores latinoamericanos. De manera similar, los talleres de formación del marco Software Carpentry/Data Carpentry, que enseñan habilidades computacionales para la ciencia de datos, se han ofrecido en español. Además, las bibliotecas digitales y los proveedores de Cursos en Línea Masivos y Abiertos (MOOC) publican ahora ediciones paralelas en español con mayor regularidad. Khan Academy y Coursera, por ejemplo, ofrecen cursos de bioinformática en español, mientras que plataformas más nuevas como Genotipia, uno

de los patrocinadores de la segunda edición del SEH2Bioinfo, ofrece programas completos en genética médica íntegramente en español, varios de los cuales están respaldados y certificados por instituciones de educación superior acreditadas.

No obstante, persisten varios desafíos. El ritmo acelerado de las actualizaciones en software y literatura de bioinformática significa que las traducciones comunitarias suelen retrasarse, convirtiendo la re-comunicación de hallazgos al español para la enseñanza o la divulgación en un paso adicional que no siempre se completa. Un segundo obstáculo es la terminología especializada: cuando las etiquetas en inglés simplemente se importan, los estudiantes deben aprender conceptos a través de nombres opacos y no descriptivos, aumentando la carga cognitiva. Para mitigar esto, algunos instructores utilizan paráfrasis descriptivas en español (p. ej., *machine learning* como "aprendizaje automatizado de patrones"), pero alcanzar un consenso comunitario en terminología requiere tiempo. Iniciativas como Enclave de Ciencia promueven la adaptación de nuevos conceptos al español en lugar de recurrir por defecto al inglés. Sin embargo, la traducción por sí sola es insuficiente; la comprensión efectiva requiere con frecuencia contextualización, ejemplos, conjuntos de datos y casos de estudio extraídos de realidades locales, que hagan el material genuinamente significativo para los estudiantes.

En el plano organizativo, los bioinformáticos hispanohablantes han conformado redes para apoyarse mutuamente y avanzar en el campo a nivel regional. Un ejemplo significativo es la Sociedad Iberoamericana de Bioinformática (SoIBio), una sociedad científica internacional fundada en 2009 con el objetivo de conectar grupos de investigación de España, Portugal y América Latina. SoIBio proporciona una plataforma de colaboración y celebra conferencias regulares (a menudo denominadas reuniones ISCB-América Latina en colaboración con la ISCB), donde el español y el portugués se hablan habitualmente junto al inglés (De Las Rivas et al., 2019). Al fomentar un sentido de comunidad, SoIBio impulsa el intercambio de materiales de formación y oportunidades de investigación en español. Además, CABANAnet ha desempeñado un papel en la formación en bioinformática con atención al idioma. Financiada por el Global Challenges Research Fund y el Chan Zuckerberg Initiative DAF, CABANAnet ha impartido talleres y becas en toda América Latina en tres áreas: enfermedades transmisibles, producción sostenible de alimentos y protección de la biodiversidad. Si bien el contenido científico ha sido universal, los instructores incorporaron frecuentemente explicaciones en español y casos de estudio localmente relevantes. El resultado ha sido una cohorte de bioinformáticos latinoamericanos que se desenvuelven con comodidad en inglés pero también pueden enseñar y orientar en español, multiplicando así la experiencia dentro de sus países de origen (Campos-Sánchez et al., 2024).

Las disparidades socioeconómicas y las barreras lingüísticas se refuerzan mutuamente en la bioinformática latinoamericana. La financiación limitada y desigual para infraestructura básica, computación de alto rendimiento, almacenamiento seguro y apoyo especializado empuja a muchas instituciones a depender de alianzas con centros mejor dotados de recursos, frecuentemente anglófonos. Estas colaboraciones amplían la capacidad técnica, pero como suelen operar en inglés, pueden relegar el español en la coordinación diaria y la toma de decisiones, concentrando el poder comunicativo en quienes tienen mayores habilidades en inglés.

El dominio del inglés en América Latina está fuertemente estratificado por el nivel socioeconómico, con una instrucción de calidad concentrada en instituciones privadas y hogares de ingresos más altos, lo que a su vez amplía las brechas académicas y profesionales (Beltrão et al., 2021; Fernández et al., 2023). Un ejemplo de esta dinámica puede observarse en Colombia, donde entre los estudiantes de doctorado en biología, un mayor nivel socioeconómico está asociado a una mayor competencia en inglés y a mayores oportunidades profesionales (Ramírez-Castañeda, 2020). Sin vías robustas en español para la formación, la documentación, los flujos de trabajo y la difusión, el campo corre el riesgo de excluir a investigadores científicamente capaces cuya principal limitación es el inglés avanzado, reproduciendo así inequidades estructurales socioeconómicas en regiones con menos recursos (Alhamami, 2023; Turba et al., 2026).

3.1 El panorama del ISCB-SC y el escenario actual

En 2004, el ISCB-SC fue fundado oficialmente, consolidando su posición como la principal comunidad global para estudiantes, postdoctorandos e investigadores en etapas tempranas en los campos de la bioinformática y la biología computacional. Para fomentar una mayor interacción entre estudiantes y científicos emergentes en estos campos, el ISCB-SC creó el programa de Grupos Regionales de Estudiantes (RSGs) en 2006. Esta iniciativa fue diseñada para facilitar la coordinación de actividades específicas que atiendan las necesidades tanto de estudiantes como de jóvenes profesionales en bioinformática dentro de sus respectivas comunidades locales. Gracias a su alcance internacional, el programa de RSGs ha catalizado el establecimiento de múltiples redes y colaboraciones entre comunidades geográficamente distantes (Shome et al., 2019).

El ISCB-SC se está expandiendo rápidamente, con nuevos RSGs formándose en todo el mundo; 10 de ellos están radicados en países hispanohablantes (Figura 1), lo que representa aproximadamente el 40% de todos los grupos regionales (Eranti et al., 2023). En consecuencia, el español es el segundo idioma de trabajo más utilizado en la web, después del inglés. Sin embargo, la mayoría de los eventos insignia continentales organizados por el ISCB-SC se llevan a cabo principalmente en este idioma. Si bien esto promueve la colaboración internacional y una mayor difusión del conocimiento, también crea una barrera lingüística para los no nativos, especialmente para estudiantes e investigadores en etapas tempranas.

El primer RSG hispanohablante en establecerse fue el RSG-España en 2010, seguido por el RSG-Argentina en 2012 (Macintyre et al., 2013). En 2014, el liderazgo latinoamericano tomó la iniciativa de organizar el primer Simposio Latinoamericano del ISCB-SC (LA-SCS) (Parra et al., 2015), un evento que se ha celebrado cada dos años desde entonces (Parra et al., 2015; Monzon et al., 2017; Parisi et al., 2019; Castillo-Vilcahuaman et al., 2020). El LA-SCS marcó un hito en las comunicaciones internacionales en América Latina y sirvió como catalizador para la formación de nuevos RSGs hispanohablantes en la región. Desde 2015,

los RSGs hispanohablantes se han expandido de manera sostenida (Figura 1), y se espera la incorporación de grupos hispanohablantes adicionales en los próximos años, lo que ampliará y fortalecerá aún más esta red colaborativa.

Además, desde 2010, la comunidad hispanohablante del ISCB-SC ha organizado una serie de simposios estudiantiles, por ejemplo, la reunión inaugural en Málaga (2010) como pre-sesión de las Jornadas de Bioinformática coorganizadas con Portugal y el RSG-Norte de África, seguida por ediciones en Barcelona (2012) y Sevilla (2014) (Rivero et al., 2022). Posteriormente, el RSG-Argentina marcó un hito regional con el primer Simposio Argentino de Jóvenes Investigadores en Bioinformática (SAJIB) en Buenos Aires (2016), el primer evento nacional organizado por un RSG hispanohablante íntegramente en español. Se realizó como satélite de la conferencia anual de la Asociación Argentina de Bioinformática y Biología Computacional (A2B2C), llevada a cabo en inglés (Parra et al., 2016). Cabe destacar que, en 2021, el RSG-Ecuador y el RSG-Venezuela organizaron conjuntamente el primer evento virtual cooperativo del ISCB-SC íntegramente en español, diseñado expresamente para responder a los desafíos planteados por la pandemia de COVID-19 (Ayala-Ruano et al., 2022).

Todas estas actividades pusieron de manifiesto la necesidad y el deseo de eventos colaborativos en idiomas distintos al inglés, orientados a facilitar la incorporación de estudiantes e investigadores en etapas tempranas a la comunidad profesional y a la práctica de reuniones científicas, así como a promover el avance de la bioinformática y la biología computacional en países no anglófonos donde el ISCB y el ISCB-SC tienen presencia.

3.2 Barreras y oportunidades

El ISCB-SC es consciente de que el idioma puede abrir puertas o mantenerlas silenciosamente cerradas. Encuestas realizadas en América Latina revelan que un tercio de los jóvenes biólogos rechaza conferencias internacionales porque las presentaciones en inglés son obligatorias, y muchos gastan sumas equivalentes a la mitad de una beca doctoral solo para que sus artículos sean editados en un inglés impecable (Ramírez-Castañeda, 2020). Estas cifras subrayan cómo el dominio de una sola lengua amplifica las divisiones económicas preexistentes y margina a científicos prometedores mucho antes de que sus ideas lleguen a la etapa de revisión por pares. Sin embargo, la propia estructura de los RSGs hispanohablantes demuestra que la inclusión no tiene que esperar a cambios sistémicos más amplios: puede comenzar en la lengua materna de los estudiantes.

Dentro de los RSGs, los investigadores en etapas tempranas tienen la oportunidad de ensayar cada faceta de la vida científica primero en su lengua materna. Como la audiencia comparte referencias culturales y expresiones idiomáticas, la retroalimentación es directa y refuerza la confianza: los presentadores perfeccionan sus argumentos sin la carga cognitiva de traducir en tiempo real. El entorno se convierte así en un laboratorio para el desarrollo de habilidades blandas: los voluntarios aprenden a redactar resúmenes, presidir simposios, responder preguntas difíciles, negociar patrocinios, redactar comunicados de prensa y orientar a los más jóvenes, todo ello mientras practican la precisión retórica que exige la comunicación científica. Solo tras múltiples iteraciones muchos participantes dan el salto al inglés, equipados con un relato pulido y una presencia escénica capaz de resistir el escrutinio de una sala de conferencias internacional. Así, el español no es una muleta sino un andamiaje que permite que las ideas alcancen su máxima altura antes de prepararse para el escenario global.

Los beneficios se extienden mucho más allá de las carreras individuales. Dado que las charlas de los RSGs suelen transmitirse en línea y luego resumirse, su contenido científico ha podido reintegrarse en aulas y redes sociales de divulgación científica en toda América Latina y España, mostrando a estudiantes de secundaria, académicos y potenciales responsables de políticas que la bioinformática también puede hablar su lengua. Las cadenas de mentoría se forman de manera natural: los miembros más experimentados que ya han dado el salto a conferencias y revistas en inglés orientan a los recién llegados en la redacción de solicitudes de financiamiento y resúmenes de conferencias, sin dejar de enfatizar el valor de publicar resultados localmente relevantes en medios hispanohablantes. De este modo, cada RSG se convierte tanto en un trampolín hacia la red internacional del ISCB como en un conducto de vuelta al espacio público hispanohablante. Desde la perspectiva del ISCB-SC, este flujo recursivo (incubación local, presentación global, reinversión local) refuerza la inclusividad científica en la que la diversidad lingüística enriquece, en lugar de fragmentar la comunidad, conduciendo a la integración global.

4. SEH2Bioinfo: un modelo de ciencia inclusiva y colaborativa en español

4.1 Orígenes, objetivos y crecimiento

SEH2Bioinfo es una iniciativa internacional única que promueve el uso del español como lengua científica. Organizado por los RSGs del ISCB-SC de países hispanohablantes, el evento tiene como objetivo superar las barreras lingüísticas en la ciencia, específicamente en la biología computacional y la bioinformática, y facilitar el acceso equitativo al conocimiento en las regiones hispanohablantes.

La primera edición, celebrada en junio de 2022 (SEH2Bioinfo, 2022), reunió a 581 participantes, convirtiéndose en el evento multi-RSG más grande de la historia del ISCB-SC. Tres años después, en abril de 2025, la segunda edición (SEH2Bioinfo, 2025) congregó a 929 asistentes de Iberoamérica, Europa, América del Norte y Asia, evidenciando un crecimiento significativo tanto en tamaño como en alcance geográfico (Figura 2). La distribución geográfica de los participantes en la Figura 2 también pone de relieve la importancia estratégica de la diáspora científica hispanohablante (Meyer, 2001). Si bien la mayoría de los asistentes estaban radicados en instituciones iberoamericanas, la presencia de investigadores en países no hispanohablantes, especialmente en Europa, subraya el papel del español como complemento profesional y emocional del inglés para los científicos que trabajan en el extranjero. Sin embargo, la escasa participación de investigadores en Estados Unidos resultó inesperada, dado el significativo número de científicos hispanos en las instituciones académicas norteamericanas. Esta ausencia sugiere una brecha en las estrategias de alcance actuales y refuerza la necesidad de una vinculación específica con las

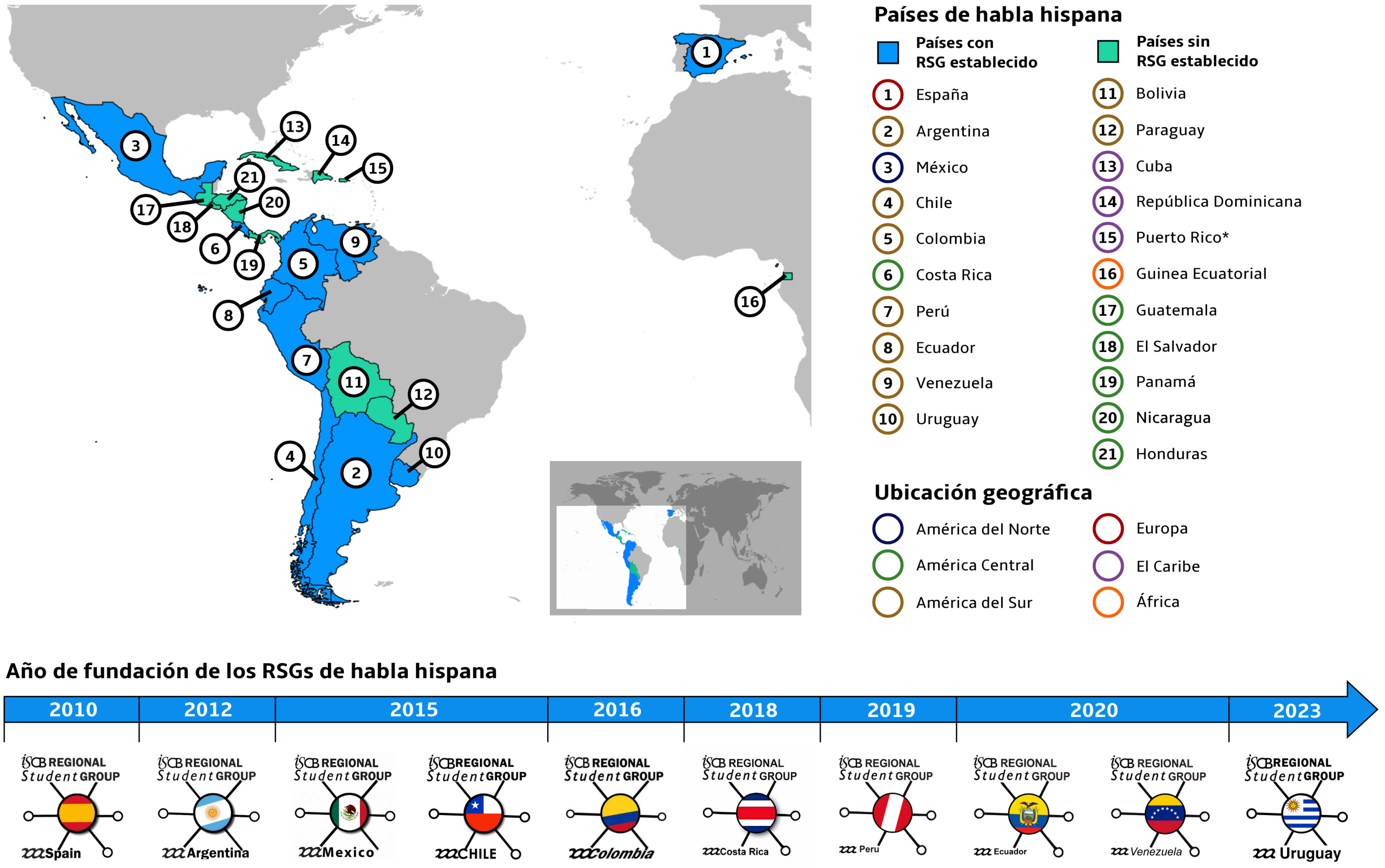

Figura 1 Evolución y distribución geográfica de los RSG hispanohablantes. El mapa (arriba) identifica los países de habla hispana con (azul) y sin (verde) RSG establecidos dentro del ISCB-SC. La línea de tiempo (abajo) ilustra los años de fundación de los diversos grupos, desde 2010 hasta 2023. *Puerto Rico se incluye como parte de la comunidad bioinformática hispanohablante debido al predominio del idioma español, aunque es un territorio no incorporado de los Estados Unidos.

sociedades científicas hispanas en países no hispanohablantes. Fortalecer estos vínculos será crucial para posicionar el español no como sustituto, sino como herramienta complementaria que fomente la colaboración y la equidad lingüística dentro de la comunidad científica global. Este progreso refleja un interés global creciente en iniciativas científicas desarrolladas en español y destaca el valor del simposio como modelo de inclusión lingüística y colaboración internacional. El perfil diverso de los participantes, desde estudiantes de pregrado, maestría y doctorado hasta investigadores en etapas tempranas y profesionales, demuestra el potencial del español como herramienta integradora para la nueva generación de científicos en biología computacional y bioinformática.

4.2 Formato, actividades y organización

El SEH2Bioinfo se llevó a cabo en un formato completamente virtual y gratuito, facilitando la interacción directa y el establecimiento de redes entre autores y asistentes en un entorno en línea inmersivo. El programa incluyó conferencias de oradores principales (Tabla 1), así como presentaciones orales, charlas relámpago y sesiones de pósters a cargo de estudiantes e investigadores en etapas tempranas, todo en español, fortaleciendo así la inclusión lingüística y el intercambio científico.

Durante la primera edición se lanzó el Programa de Mentoría en Bioinformática en Español, pionero en su tipo, con 86 mentees y 23 mentores de distintos países hispanohablantes y algunos no hispanohablantes, sentando las bases para interacciones a largo plazo. En 2025 se introdujo el concurso "Tu presentación en 2 minutos" para mostrar la investigación en un formato conciso y accesible. Todas las contribuciones pasaron por revisión por pares realizada por un comité de estudiantes de doctorado e investigadores en etapas tempranas, utilizando una rúbrica estandarizada basada en originalidad, solidez metodológica, reproducibilidad y relevancia. Los temas abordados incluyeron aprendizaje automático, transcriptómica de célula única, genómica funcional, biología evolutiva, biología estructural, análisis multi-ómico y modelado de redes biológicas, siendo la mayoría de los participantes estudiantes de grado o posgrado e investigadores en etapas tempranas de entre 20 y 35 años.

Ambas ediciones incorporaron actividades lúdicas y culturales, como una sala de escape temática y un cuestionario sobre países hispanohablantes, fomentando un ambiente igualitario y colaborativo al tiempo que se fortalecía la identidad cultural compartida de la comunidad. En la segunda edición se crearon listas de reproducción en Spotify para cada país hispanohablante, permitiendo a los participantes explorar la diversidad musical de los países involucrados, encontrar

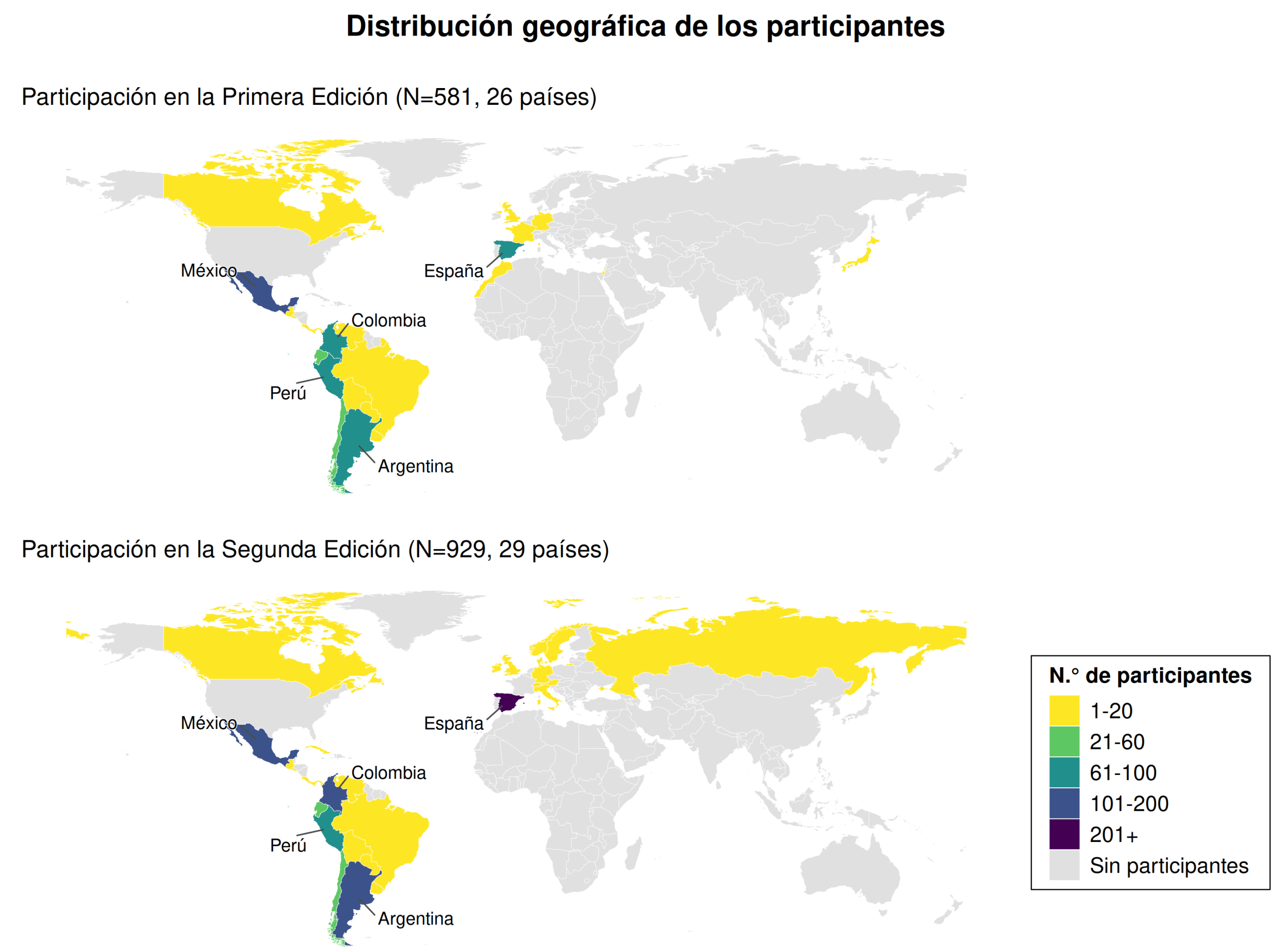

Figura 2 Distribución geográfica de los participantes en las ediciones del SEH2Bioinfo. Los datos corresponden a la primera (arriba) y segunda (abajo) edición del simposio. La representación por país se basa en la afiliación institucional. La escala de colores categoriza el número de participantes por país; el gris claro indica ausencia de participación. Los países con un volumen significativo de participación ($n > 60$) en ambas ediciones están etiquetados explícitamente.

inspiración durante las sesiones de trabajo y reforzar los vínculos culturales y emocionales.

El SEH2Bioinfo recibió apoyo de sociedades científicas y patrocinadores privados, incluyendo el ISCB-SC, el Instituto Nacional de Bioinformática español (INB ELIXIR-ES), la Sociedad Chilena de Bioinformática (SCB), la A2B2C, y las empresas Dreamgenics, Genotipia y Vakmo Technologies. Este apoyo pone de relieve que la colaboración entre entidades científicas y el sector privado es clave para sostener eventos inclusivos y de alta calidad, fortalecer los vínculos de los jóvenes científicos con redes internacionales y legitimar el impacto del simposio dentro del ecosistema científico hispanohablante.

El SEH2Bioinfo fue organizado de manera colaborativa por los RSGs hispanohablantes activos (Tabla 1), con subcomités especializados que trabajaron a través de plataformas digitales. Se implementaron estrategias de comunicación inclusiva, conciencia intercultural y trabajo en equipo para gestionar la diversidad cultural y los diferentes niveles de experiencia de los participantes. Aunque los eventos se desarrollaron íntegramente en español, la comunicación con el Equipo Ejecutivo del ISCB-SC y los colaboradores externos se realizó en inglés, adoptando un enfoque bilingüe adaptativo. Los canales oficiales y las cuentas de redes sociales publicaron contenido en ambos idiomas para ampliar la visibilidad, mientras que los aspectos técnicos, como la conectividad, la compatibilidad de plataformas y la gestión del público, se abordaron mediante ensayos, soporte técnico y planificación de contingencias.

En conjunto, la Tabla 1 resume la evolución del simposio entre 2022 y 2025, mostrando cómo la colaboración transatlántica entre países hispanohablantes, con sus variantes lingüísticas y estilos de comunicación, junto con las diferencias en los niveles académicos del comité organizador, la experiencia en gestión de eventos y la creciente participación de los asistentes, contribuyó a consolidar el SEH2Bioinfo como referente de la bioinformática en español.

4.3 El desafío de fomentar la participación en bioinformática

A partir de las encuestas realizadas durante las dos ediciones del SEH2Bioinfo, con un total de 1.510 participantes (Figura 3), emergen datos significativos sobre las barreras lingüísticas que enfrenta la comunidad hispanohablante de bioinformáticos y biólogos computacionales. En el escenario actual, la figura muestra una comunidad que opera bajo el dominio del inglés pero con un nivel de competencia desigual: aproximadamente el 61% de los encuestados se ubica en niveles básico/intermedio, mientras que solo alrededor del 39% declara un nivel avanzado o casi nativo. Esta brecha tiene consecuencias evidentes. Alrededor del 68% ha evitado presentar en eventos científicos por inseguridad con su inglés, y el 55% siente que las barreras lingüísticas han limitado sus colaboraciones internacionales. Una ajustada mayoría (52%) cree que su trabajo tendría mayor impacto si se presentara en su lengua materna, aunque solo el 42% ha llegado a presentar o publicar en ella.

Las preferencias son inequívocas. Un abrumador 91% desea más espacios para presentaciones no en inglés en reuniones internacionales. Cuando deben usar el inglés, la confianza se inclina hacia el texto: el 57% se siente más cómodo escribiendo que hablando (27%), con un 17% que no se siente seguro en ninguno de los dos, lo que pone de relieve la comunicación oral como un punto de especial dificultad.

En conjunto, los datos apuntan a la necesidad de intervenciones prácticas a corto plazo, como sesiones temáticas y de pósters multilingües; resúmenes y diapositivas bilingües; subtitulación o interpretación en tiempo real; opciones para presentar en español con materiales en inglés; y formación para presentaciones en público, que redujeran la autoexclusión, ampliaran la colaboración y alinearan mejor la difusión con la realidad lingüística de la comunidad. El SEH2Bioinfo actúa como agente concreto de cambio al implementar estas medidas. Como foro inclusivo donde los participantes pueden comunicarse con claridad y confianza en español, el simposio reencuadra el idioma como facilitador en lugar de barrera y sirve de modelo de políticas que eventos más grandes podrían adoptar. De este modo, funciona como infraestructura inmediata de equidad y como catalizador de una profunda evolución del ecosistema científico.

4.4 Impacto y proyecciones del SEH2Bioinfo

El SEH2Bioinfo, en sus dos ediciones, se ha convertido en un referente dentro de la comunidad hispanohablante de bioinformática. Su impacto se refleja en su crecimiento sostenido, el fortalecimiento de una red regional de jóvenes científicos y el uso activo del español para la formación, la comunicación y la colaboración científica. Su alcance geográfico abarca no solo América Latina y España, sino también a hispanohablantes nativos que residen en regiones no hispanohablantes. Esta iniciativa también despierta un gran interés entre instituciones de todo el mundo comprometidas con modelos de comunicación inclusivos. La participación de personas provenientes de aproximadamente 200 instituciones públicas y privadas de diversos países respalda la legitimidad y la proyección internacional del evento.

En comparación con otras conferencias internacionales de bioinformática que suelen orientarse a comunidades globales y exigen elevadas cuotas de inscripción, el SEH2Bioinfo se distingue por su énfasis en el español y su accesibilidad virtual. Mientras que los eventos tradicionales limitan la participación de estudiantes de regiones económicamente desfavorecidas, el SEH2Bioinfo mantiene un modelo gratuito que elimina las barreras económicas y promueve la equidad. Con su formato interactivo, que combina actividades dinámicas y mesas redondas multirregionales, se ha consolidado como un evento innovador dentro de la comunidad científica hispanohablante. Su crecimiento y diversidad institucional lo posicionan como una plataforma clave para promover una ciencia accesible, inclusiva y global desde esta comunidad en los próximos años.

La próxima edición del SEH2Bioinfo está planificada para 2028, idealmente de forma presencial para maximizar el establecimiento de redes y la mentoría. El objetivo es reunir a los líderes más destacados de la bioinformática en países hispanohablantes, desde investigadores principales consolidados hasta estudiantes, en particular quienes han sido promotores activos de la equidad y la inclusión en el campo. Se pondrá especial énfasis en impulsar a jóvenes líderes con capacidad para formar a una nueva generación de bioinformáticos sustentados en la excelencia científica, la responsabilidad social y el enriquecimiento cultural. El programa se concibe combinando conferencias de alto nivel con formación práctica, clínicas de mentoría y diálogos sobre política científica, consolidando el papel del SEH2Bioinfo como catalizador de una comunidad hispanohablante de bioinformática más inclusiva y conectada globalmente. Para alcanzar estos objetivos, el simposio tiene la intención de adoptar estrategias prácticas de equidad lingüística inspiradas en comunidades internacionales exitosas. Siguiendo modelos como PyLadies Global, evaluaremos la implementación de herramientas de subtitulación en tiempo real y traducción simultánea impulsadas por inteligencia artificial como parte de nuestra infraestructura tecnológica. Estas acciones buscan fomentar el "multilingüismo receptivo" (ten Thije and Zeevaert, 2007), un marco que aprovecha la inteligibilidad mutua para garantizar que el contenido técnico fundamental sea accesible a todos los asistentes a pesar de las posibles barreras lingüísticas. Si bien nuestro enfoque principal sigue siendo el fortalecimiento de la red hispanohablante para promover la equidad lingüística en la ciencia, no descartamos la posibilidad de incorporar formatos multilingües (p. ej., español-portugués-inglés) en futuras ediciones presenciales o híbridas, siguiendo referentes exitosos como la iniciativa Allbiotech en el fomento de la colaboración científica iberoamericana.

5. Perspectivas de futuro: el español como lengua científica eficaz e inclusiva

El español se encuentra hoy en el umbral de un papel más prominente en el intercambio científico, con un impulso que se consolida en múltiples frentes. Los editores están transformando silenciosamente el panorama editorial: un número creciente de revistas y conferencias alienta o exige a los

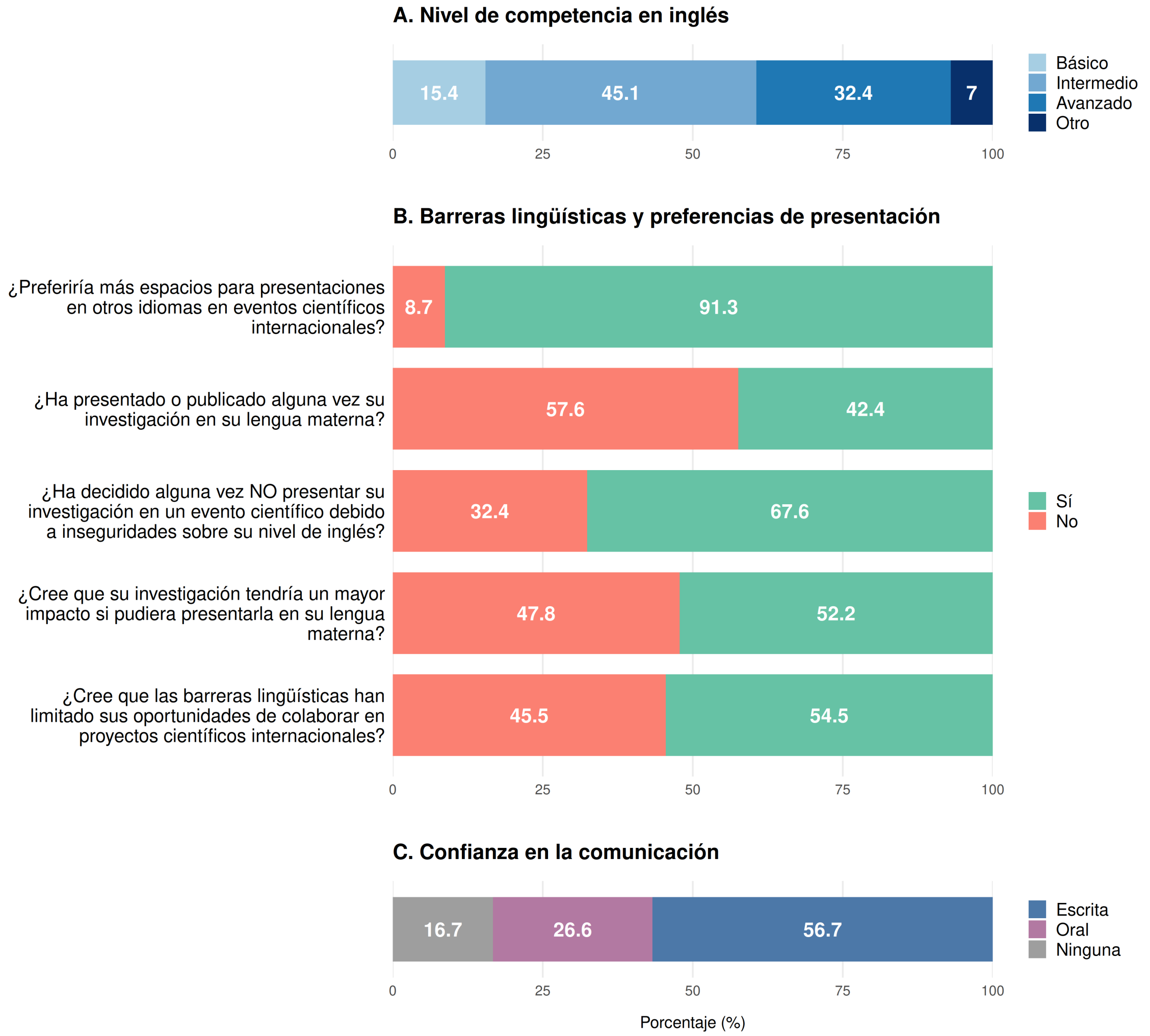

Figura 3 Análisis porcentual de las barreras lingüísticas y nivel de inglés autoinformado. Los datos fueron recolectados a través de encuestas realizadas durante las dos primeras ediciones del evento SEH2Bioinfo. **A.** Distribución de los niveles de competencia en inglés entre los participantes. **B.** Impacto de las barreras lingüísticas en la trayectoria académica y preferencias de presentación en eventos internacionales. **C.** Niveles comparativos de confianza según el modo de comunicación. Todos los valores se expresan como porcentajes de la muestra total ($N = 1.510$).

autores incluir un resumen divulgativo o un abstract en español junto al texto tradicional en inglés. Emerging Themes in Epidemiology, por ejemplo, ya traduce los resúmenes a varios idiomas. SciELO de Brasil y RedALyC de México llevan dos décadas construyendo reconocidos catálogos de acceso abierto (Packer et al., 2014; Becerril-García and Aguado-López, 2019). Además, los organismos de financiación están explorando nuevas estrategias para elevar su prestigio, que van desde el apoyo a traducciones profesionales hasta la incorporación de métricas de citación regionales, con el objetivo de garantizar que las publicaciones en español alcancen un impacto comparable al de las publicaciones en inglés (Vessuri et al., 2014; UNESCO, 2021).

El avance tecnológico promete acelerar ese cambio cultural. Los sistemas de traducción automática específicamente ajustados a la prosa científica se aproximan a un nivel en el que un artículo escrito en inglés puede convertirse casi instantáneamente al español con terminología propia de la disciplina. Los científicos podrían llegar a enviar un artículo en español a una revista no hispanohablante y confiar en traducciones automáticas para la revisión por pares y la publicación. Proyectos piloto ya han recopilado preprints traducidos en repositorios abiertos, generando de forma colectiva un corpus multilingüe de ciencia. En un futuro no muy lejano, los ponentes de conferencias podrían dirigirse a su audiencia en español mientras una transmisión de subtítulos por inteligencia artificial vierte la charla al inglés (o al idioma que prefiera el participante). Tales herramientas redistribuyen la carga comunicativa: los angloparlantes podrán leer pronto investigación en español en traducción fluida, en lugar de esperar que cada científico hispanohablante domine primero el inglés.

Los gobiernos y las instituciones están dando peso formal a estas tendencias. La Fundación Española para la Ciencia y la Tecnología (FECYT) ha comenzado a exigir que los entregables de los proyectos financiados incluyan un resumen en lenguaje sencillo en español, y la agencia financia medios

como la Agencia SINC para garantizar que la investigación nacional llegue al público en español. En toda América Latina, los festivales de la Semana de la Ciencia introducen a los escolares en demostraciones de laboratorio en español, formando a una nueva generación con confianza para hablar de temas CTIM en su lengua materna. En el escenario multilateral, la Recomendación de la UNESCO de 2021 sobre Ciencia Abierta llama explícitamente a la difusión multilingüe, y el Consejo Internacional de la Ciencia se ha hecho eco de esta postura, señalando que la equidad lingüística ya no es una preocupación marginal sino parte de la doctrina central de la política científica (UNESCO, 2021).

La energía de la comunidad amplifica estos esfuerzos. Los investigadores en etapas tempranas consideran la divulgación bilingüe como un deber moral y una marca profesional. Proyectos como CABANAnet, SolBio y Scientia en Boise State demuestran que traductores y comunicadores voluntarios pueden construir plataformas de alto impacto que normalicen el español y el portugués en salud, agricultura y bioinformática. A medida que estos científicos acceden a consejos editoriales y comités de financiación, llevan consigo la convicción de que el bilingüismo en revistas y las colaboraciones Sur-Sur deben ser una práctica estándar. Redes como el Programa Iberoamericano de Ciencia y Tecnología para el Desarrollo (CYTED) ya coordinan clusters temáticos entre España y América Latina (Saigí-Rubió et al., 2017); un futuro consorcio podría publicar un estudio de referencia sobre la biodiversidad amazónica en inglés y español en paralelo, sentando un precedente para la producción académica global orientada a las partes interesadas locales.

Los campos interdisciplinarios y computacionales pueden ser los primeros en cosechar los dividendos. Los pipelines bioinformáticos que procesan datos de salud pública pierden valor si los epidemiólogos locales no pueden leer la interfaz; los desarrolladores han comenzado a incorporar equipos de internacionalización (i18n) para traducir cadenas de software y documentación (Ouhbi et al., 2017). La cultura del código abierto encaja perfectamente con la inclusión lingüística: los programadores voluntarios pueden traducir y adaptar herramientas con la misma facilidad con la que incorporan nuevas funcionalidades. La educación en línea sigue la misma trayectoria. Khan Academy, Coursera, Genotipia y Flyteek han ampliado notablemente sus catálogos en español, esbozando MOOCs de bioinformática impartidos desde el primer día en español (Ding et al., 2014). Mientras tanto, la Wikipedia en español, con más de un millón de entradas, se enriquece mediante maratones de edición que reclutan expertos para mejorar las páginas científicas (Sélem-Mojica et al., 2024).

Estos avances no eliminan todos los obstáculos. El inglés seguirá siendo la moneda de las publicaciones al nivel del Nobel en un futuro previsible, y producir contenido en dos idiomas exige tiempo, dinero y apoyo institucional sostenido. El impulso al español debe evitar crear un silo aislado; el resultado ideal es un científico bilingüe que pueda publicar en inglés para alcance global y al mismo tiempo relacionarse con colegas y públicos en español. La verdadera equidad debe reconocer también la importancia de las lenguas indígenas (p. ej., quechua, aimara, guaraní), que en muchos países latinoamericanos son tan culturalmente e históricamente significativas como el español (Hornberger, 2006). Dado el peso demográfico de más de 500 millones de hablantes nativos, los rápidos avances en traducción asistida por inteligencia artificial (Mager et al., 2023) y la energía sostenida de una nueva generación de investigadores, el español está preparado para pasar de los márgenes del discurso científico a una asociación complementaria con el inglés. En este emergente panorama multilingüe, el español aporta perspectiva regional, contexto cultural y mayor alcance público, haciendo el conocimiento científico genuinamente más accesible a las comunidades a las que pretende servir.

Conclusión

El inglés seguirá desempeñando un papel central en la comunicación científica global. Sin embargo, nuestro análisis sugiere que esta centralidad no tiene por qué excluir espacios complementarios y cuidadosamente diseñados en otras lenguas que amplíen la participación y mejoren la formación, especialmente para investigadores en etapas tempranas en campos interdisciplinarios y en rápida evolución como la bioinformática. Al situar el uso del español en un panorama histórico y contemporáneo, y al examinar dos ediciones del SEH2Bioinfo, ofrecemos evidencia de que los formatos lingüísticamente inclusivos pueden coexistir con el compromiso internacional y, en última instancia, fortalecerlo.

A lo largo de las ediciones de 2022 y 2025 del SEH2Bioinfo (581 y 929 asistentes, respectivamente) y de una encuesta a 1.510 participantes, observamos fricciones lingüísticas persistentes que afectan las tasas de presentación, la colaboración y la autoeficacia. El diseño del simposio, el español como lengua de trabajo, la selección de resúmenes por revisión por pares, la mentoría estructurada y el establecimiento interactivo de redes, parece mitigar algunas de estas barreras preservando los estándares académicos. En términos prácticos, el SEH2Bioinfo funciona tanto como infraestructura de equidad a corto plazo: (i) un espacio donde los participantes pueden pensar y presentar en su lengua más sólida; y (ii) como puente hacia foros globales, a través de materiales bilingües y actividades de desarrollo profesional.

Las implicaciones van más allá de un único evento o idioma. En bioinformática, donde las aplicaciones en salud pública y sistemas agroalimentarios dependen de la implementación local, la formación multilingüe, la documentación y el uso de casos de estudio pueden mejorar la relevancia y la adopción de herramientas y metodologías. El modelo SEH2Bioinfo, que combina el uso del idioma local para el aprendizaje y la construcción de comunidad con la difusión en inglés, podría aplicarse a otros contextos y complementar iniciativas de ciencia abierta, esfuerzos de fortalecimiento de capacidades y la colaboración Sur-Sur.

Con la debida cautela, sugerimos varios pasos modestos y respaldados por evidencia que las instituciones y sociedades hispanohablantes podrían considerar: resúmenes, sesiones temáticas y de pósters multilingües; subtitulación o interpretación en tiempo real; opciones para presentar en español con materiales en inglés; microbecas para edición y traducción; y reconocimiento de la mentoría y divulgación bilingüe. Tales medidas son de bajo costo en relación con su potencial para reducir la autoexclusión y alinear las prácticas de difusión con las realidades lingüísticas de los investigadores.

En conjunto, nuestras experiencias respaldan una visión complementaria de la práctica lingüística en la ciencia: el inglés sigue siendo indispensable para el alcance global, mientras que los entornos en español pueden mejorar la equidad, la calidad de la formación y el impacto regional. Esperamos que esta evidencia anime a revistas, sociedades y organismos de financiación a pilotear medidas multilingües proporcionadas y basadas en datos que preserven las eficiencias de un idioma común al mismo tiempo que amplían el círculo de quienes pueden participar y beneficiarse de la bioinformática contemporánea.

Finalmente, expresamos nuestro sincero agradecimiento al ISCB y al ISCB-SC por su compromiso continuo con el fomento de comunidades científicas diversas y globalmente conectadas. Su visión y apoyo han hecho posibles iniciativas como el SEH2Bioinfo, demostrando que la inclusión y la excelencia pueden avanzar de la mano.

Agradecimientos

Reconocemos a los patrocinadores que hicieron posible el SEH2Bioinfo, a los organizadores de los distintos RSGs hispanohablantes y a los colaboradores que contribuyeron a la difusión, la logística y otras tareas esenciales para su realización. G.O.-O. agradece el apoyo financiero del Proyecto Postdoctoral FONDECYT N.º 3250793. G.O.-O. expresa también su gratitud a Valentina Luza: "¡Que su pasión por la comunicación científica siga inspirando a nuevas generaciones, incluido yo mismo, hasta el infinito y más allá!" R.G.P. agradece al sistema de educación universitaria pública argentina como elemento de ascenso social y democratización de las oportunidades profesionales.

Conflicto de intereses

Ninguno declarado.

Financiamiento

Los simposios SEH2Bioinfo contaron con el apoyo del Consejo Estudiantil de la Sociedad Internacional de Biología Computacional (ISCB-SC), el Instituto Nacional de Bioinformática español (INB ELIXIR-ES), la Sociedad Chilena de Bioinformática (SCB) y la Asociación Argentina de Bioinformática y Biología Computacional (A2B2C). El patrocinio del evento también fue provisto por Dreamgenics, Genotipia y Vakmo Technologies. Las entidades mencionadas proporcionaron apoyo financiero para los eventos, pero no tuvieron ningún papel en el diseño del estudio, la recopilación, el análisis y la interpretación de los datos, ni en la redacción del manuscrito.

Material suplementario

Traducción: Este artículo es una traducción al español del artículo original publicado en inglés, el cual puede consultarse a través del DOI: `https://doi.org/10.1093/bioadv/vbag182`. Tras su aceptación, esta versión en español se depositará en Zenodo para garantizar su acceso abierto.

Disponibilidad de datos: Los datos que respaldan este artículo, incluidos los libros de resúmenes de la primera y segunda edición de los simposios SEH2Bioinfo, están disponibles en Zenodo en `https://doi.org/10.5281/zenodo.6603621` y `https://doi.org/10.5281/zenodo.15446570`, respectivamente.

Referencias

Alhamami M. Inequity, inequality, and language rights in English as a medium of instruction programs. *Evaluation and Program Planning*, **99**, 102297, 2023.

Amano T, González-Varo JP, and Sutherland WJ. Languages are still a major barrier to global science. *PLoS Biology*, **14**(12), e2000933, 2016.

Amano T, Ramírez-Castañeda V, Berdejo-Espinola V, Borokini I, Chowdhury S, Golivets M, González-Trujillo JD, Montaño-Centellas F, Paudel K, White RL, et al. The manifold costs of being a non-native English speaker in science. *PLoS Biology*, **21**(7), e3002184, 2023.

Ammon U. *The dominance of English as a language of science: Effects on other languages and language communities*, volume 84. Walter de Gruyter, 2001.

Ammon U. Linguistic inequality and its effects on participation in scientific discourse and on global knowledge accumulation. *Applied Linguistics Review*, **3**(2), 2012.

Ayala-Ruano S, Hernandez F, Ortega A, Infante D, Carrascal D, Sánchez-Luquez K, and Puche-Quiñonez R. Highlights of the 1st Ecuadorian-Venezuelan Symposium of Young Researchers in Bioinformatics (1SEVJIB). *F1000Research*, **11**, 1086, 2022.

Becerril-García A and Aguado-López E. The end of a centralized open access project and the beginning of a community-based sustainable infrastructure for Latin America. *Connecting the Knowledge Commons form Projects to Sustainable Infrastructure*, pages 41–55, 2019.

Beltrão KI, Mandarino M, Megahós RS, and Pedrosa MGF. Effects of socioeconomic status on the use of written language: does it extend into Brazilian higher education? *Ensaio: Avaliação e Políticas Públicas em Educação*, **29**(112), 618–643, 2021.

Boyle S, Rizzo KL, and Taylor JC. Reducing language barriers in science for students with special educational needs. *Asia-Pacific Science Education*, **6**(2), 364–387, 2020.

Campos-Sánchez R, Willis I, Gopalasingam P, López-Juárez D, Cristancho M, Brooksbank C, and Consortium C. The CABANA model 2017–2022: research and training synergy to facilitate bioinformatics applications in Latin America. In *Frontiers in education*, volume 9, page 1358620. Frontiers Media SA, 2024.

Castillo-Vilcahuaman C, Valdivia C, Osorio-Mogollón C, Silva-Andrade C, Puche R, Ayala-Ruano S, Cuesta-Astroz Y, and Parra RG. 4th ISCB Latin American Student Council Symposium: a virtual and inclusive experience during COVID-19 times. *F1000Research*, **9**, ISCB–Comm, 2020.

Claret J. Schools, scientific institutions, and the Spanish "brain drain" (1833–2023). In *The Routledge Hispanic Studies Companion to Twentieth and Twenty-First Century Spain*, pages 261–273. Routledge, 2025.

De Las Rivas J, Bonavides-Martínez C, and Campos-Laborie FJ. Bioinformatics in Latin America and SolBio impact, a tale of spin-off and expansion around genomes and protein structures. *Briefings in bioinformatics*, **20**(2), 390–397, 2019.

Ding Y, Wang M, He Y, Ye AY, Yang X, Liu F, Meng Y, Gao G, and Wei L. "bioinformatics: introduction and methods," a bilingual massive open online course (MOOC) as a new example for global bioinformatics education. *PLoS computational biology*, **10**(12), e1003955, 2014.

Ehrman ME. *Understanding second language learning difficulties*. Sage, 1996.

Eranti P, Grover A, Padilla Franzotti CL, et al. ISCB student council regional student groups: connecting young computational biologists around the Globe. In *The 31st Annual Intelligent Systems for Molecular Biology and the 22nd Annual European Conference on Computational Biology (ISMB/ECCB 2023), Lyon, France*. Zenodo, 2023. URL https://doi.org/10.5281/zenodo.8228659.

Fernández R, Pagés C, Székely M, and Acevedo I. Education Inequalities in Latin America and the Caribbean. IDB Working Paper Series IDB-WP-1538, Inter-American Development Bank, 2023.

Fransen S. Latin in a time of change: the choice of language as signifier of a new science? *Isis*, **108**(3), 629–635, 2017.

Gangui A and Ortiz EL. The scientific impact of Einstein's visit to Argentina, in 1925. *arXiv preprint arXiv:1603.03792*, 2016.

Gilakjani AP and Sabouri NB. Learners' listening comprehension difficulties in English language learning: A literature review. *English Language Teaching*, **9**(6), 123–133, 2016.

González G, Uran Landaburu L, Palopoli N, Stocchi N, Revuelta MV, Bustamante JP, Parra G, Zea DJ, Lanzarotti E, Banchero M, and Teppa E. *Vida. exe: Desafíos y aventuras de la bioinformática*. Fondo de Cultura Económica Argentina, 2022.

Hamel RE. El campo de las ciencias y la educación superior entre el monopolio del inglés y el plurilingüismo: elementos para una política del lenguaje en América Latina. *Trabalhos em Linguística Aplicada*, **52**, 321–384, 2013.

Hornberger NH. Voice and biliteracy in indigenous language revitalization: Contentious educational practices in Quechua, Guarani, and Māori contexts. *Journal of Language, Identity, and Education*, **5**(4), 277–292, 2006.

Hultgren AK. English as the language for academic publication: On equity, disadvantage and 'non-nativeness' as a red herring. *Publications*, **7**(2), 31, 2019.

Instituto Cervantes. *El Español en el mundo 2025*. Rodona Industria Gráfica, S.L., 2025. URL https://cvc.cervantes.es/lengua/anuario/anuario_25/el_espanol_en_el_mundo_anuario_instituto_cervantes_2025.pdf.

Kim R, Roberson L, Russo M, and Briganti P. Language diversity, nonnative accents, and their consequences at the workplace: Recommendations for individuals, teams, and organizations. *The Journal of Applied Behavioral Science*, **55**(1), 73–95, 2019.

López-Pérez L and Olvera-Lobo M-D. Public communication of science in Spain: a history yet to be written. *JCOM: Journal of Science Communication*, **16**(3), Y02, 2017. doi: 10.22323/2.16030502.

Macintyre G, Michaut M, and Abeel T. The regional student group program of the ISCB student council: stories from the road. *PLoS computational biology*, **9**(9), e1003241, 2013.

Mager M, Bhatnagar R, Neubig G, Vu NT, and von der Wense K. Neural machine translation for the indigenous languages of the Americas: An introduction. In *Proceedings of the Workshop on Natural Language Processing for Indigenous Languages of the Americas (AmericasNLP)*, pages 109–133, 2023.

May S. Misconceiving minority language rights: Implications for liberal political theory. In Kymlicka W and Patten A, editors, *Language Rights and Political Theory*, pages 123–152. Oxford University Press, 2003.

Meyer J-B. Network approach versus brain drain: lessons from the diaspora. *International migration*, **39**(5), 91–110, 2001.

Montgomery S. Of towers, walls, and fields: perspectives on language in science. *Science*, **303**(5662), 1333–1335, 2004.

Monzon AM, Hasenahuer MA, Mancini E, Coimbra N, Cravero F, Cáceres-Molina J, Ramírez-Sarmiento CA, Palopoli N, Meysman P, and Parra RG. Second ISCB latin american student council symposium (LA-SCS) 2016. *F1000Research*, **6**, ISCB–Comm, 2017.

Moreno AI, Rey-Rocha J, Burgess S, López-Navarro I, and Sachdev I. Spanish researchers' perceived difficulty writing research articles for English-medium journals: The impact of proficiency in English versus publication experience. *Ibérica: Revista de la Asociación Europea de Lenguas para Fines Específicos (AELFE)*, (24), 157–184, 2012. ISSN 1139-7241.

Moskaleva O and Akoev M. Non-English language publications in Citation Indexes–quantity and quality. *arXiv preprint arXiv:1907.06499*, 2019.

Moutinho S. Latin America is a leader in nonprofit open-access journals. but it struggles to give them global visibility. *Science*, **386**(6726), 1087–1089, 2024. doi: 10.1126/science.adv0401.

Ouhbi S, Fernández-Alemán JL, Carrillo-de Gea JM, Toval A, and Idri A. E-health internationalization requirements for audit purposes. *Computer methods and programs in biomedicine*, **144**, 49–60, 2017.

Packer AL, Cop N, Luccisano A, Ramalho A, and Spinak E. *SciELO: 15 years of open access: an analytic study of open access and scholarly communication*. UNESCO, París, 2014. URL https://unesdoc.unesco.org/ark:/48223/pf0000225483.

Parisi D, Olguín-Orellana GJ, Draizen EJ, Coimbra NDR, Papadopoulos N, Kirchen S, Gladbach YS, Fadra N, Fatima N, Namasivayam AA, et al. Nurturing tomorrow's leaders: The ISCB student council symposia in 2018. *F1000Research*, **8**, ISCB–Comm, 2019.

Parra RG, Simonetti FL, Hasenahuer MA, Olguin-Orellana GJ, and Shanmugam AK. Highlights from the 1st ISCB latin american student council symposium 2014. *BMC bioinformatics*, **16**(Suppl 8), A1, 2015.

Parra RG, Defelipe LA, Guzovsky AB, Monzon AM, Cravero F, Mancini E, Moreyra N, Padilla Franzotti CL, Revuelta MV, Freiberger MI, Gonzalez NN, Gonzalez GA, Orts F, Stocchi N, Hasenahuer MA, Teppa E, Zea DJ, and Palopoli N. Highlights of the 1st Argentine Symposium of Young Bioinformatics Researchers (1SAJIB) organized by the ISCB RSG-Argentina. *PeerJ Preprints*, **4**, e2494v1, 2016. doi: 10.7287/peerj.preprints.2494v1.

Parra-Perez AM, Gomez Martinez AM, Padial-Fuillerat E, Torres-Martos A, Ellson I, Martinez-Larrinaga A, Benitez-Cantos MS, Fornelino-Gonzalez R, Lopez-Dominguez R, and Garcia-Moreno A. Guía de Formación en España en

Bioinformática. *Zenodo*, 2021. doi: 10.5281/zenodo.13986151. URL https://doi.org/10.5281/zenodo.13986151.

Ramírez-Castañeda V. Disadvantages in preparing and publishing scientific papers caused by the dominance of the English language in science: The case of Colombian researchers in biological sciences. *PLoS ONE*, **15**(9), e0238372, 2020.

Rivero I, Gorines-Cordero GJ, Rubio-Rodríguez LA, Soler-Sáez I, Perpiñá-Clérigues C, García A, Monzón S, and Hernández-Beeftink T. ISCB RSG-Spain and highlights from the VIII Spanish Student Symposium in Bioinformatics and Computational Biology in 2021. *bioRxiv*, pages 2022–08, 2022.

Saigí-Rubió F, Novillo-Ortiz D, and Piette JD. Red CYTED-RITMOS: hacia la búsqueda de soluciones para fomentar la salud móvil en América Latina. *Revista Panamericana de Salud Pública*, **41**, e33, 2017.

SEH2Bioinfo. *Libro de resúmenes I Simposio de Estudiantes Hispanohablantes de Bioinformática y Biología Computacional - I SEH2Bioinfo*. Zenodo, 2022. URL https://doi.org/10.5281/zenodo.6603621.

SEH2Bioinfo. *Libro de resúmenes 2do Simposio de Estudiantes Hispanohablantes de Bioinformática y Biología Computacional - 2SEH2Bioinfo*. Zenodo, 2025. URL https://doi.org/10.5281/zenodo.15446570.

Sélem-Mojica N, Karakulak T, Anjum A, Pashkov A, Pérez-Estrada R, Enriquez-Guillén K, DeBlasio D, Ferreira-Gonzalez S, Medina-Rivera A, Rodrigo-Torres D, et al. Closing the computational biology 'knowledge gap': Spanish Wikipedia as a case study. *Bioinformatics*, **40**(Supplement_1), i11–i19, 2024.

Shome S, Parra RG, Fatima N, Monzon AM, Cuypers B, Moosa Y, Coimbra NDR, Assis J, Giner-Delgado C, Dönertaş HM, et al. Global network of computational biology communities: ISCB's Regional Student Groups breaking barriers. *F1000Research*, **8**, ISCB–Comm, 2019.

Tardy C. The role of English in scientific communication: lingua franca or Tyrannosaurus rex? *Journal of English for Academic Purposes*, **3**(3), 247–269, 2004.

ten Thije JD and Zeevaert L. *Receptive multilingualism: linguistic analyses, language policies, and didactic concepts*, volume 6. John Benjamins Publishing, 2007.

Tolmasquim AT. Science and ideology in Einstein's visit to South America in 1925. In *Einstein and the Changing Worldviews of Physics*, pages 117–133. Springer, 2011.

Turba R, Thoré ES, Bertram MG, Bridg H, Sabet SS, Gamboa M, Ríos-Orjuela JC, Takola E, Capa Salinas J, Sampaio Franco AC, et al. Global North-South science inequalities due to language and funding barriers. *Peer Community Journal*, **6**, 2026.

UNESCO. UNESCO Recommendation on Open Science. *Zenodo*, nov 2021. doi: 10.5281/zenodo.5741832. URL https://doi.org/10.5281/zenodo.5741832.

Vessuri H, Guédon J-C, and Cetto AM. Excellence or quality? impact of the current competition regime on science and scientific publishing in Latin America and its implications for development. *Current sociology*, **62**(5), 647–665, 2014.

Tabla 1. Resumen de las principales características de las ediciones 2022 y 2025 del SEH2Bioinfo.

Edición del simposio	Primera	Segunda
**RSGs organizadores**	Argentina, España, Chile, México, Venezuela, Colombia, Perú, Ecuador, Costa Rica y Uruguay[α].	
**Fechas**	2 y 3 de junio de 2022	1 y 2 de abril de 2025
**Participantes registrados**	581	929
**Presentaciones orales**	14	22
**Presentaciones de póster**	73	108
**Instituciones representadas**	163	183
**Conferencistas invitados**	Fátima Al-Shahrour (España)<br>Francisco Melo (Chile)<br>Patricia Melin (México)	Alfonso Valencia (España)<br>Cristina Marino-Buslje (Argentina)<br>Alejandra Medina-Rivera (México)
**Patrocinadores**	5	6

[α] El RSG Uruguay se incorporó como organizador únicamente para la edición 2025.
